# Comparative Physicochemical, Structural, Thermal, and Rheological Analyses of Lemon By-Product Pectin: Hot Acid-Assisted Extraction Coupled with Drying Techniques

**DOI:** 10.3390/polym18161989

**Published:** 2026-08-15

**Authors:** Daniela Magalhães, Cristina V. Rodrigues, Sérgio Sousa, Joana R. Costa, Paula Teixeira, Manuela Pintado

**Affiliations:** CBQF–Centro de Biotecnologia e Química Fina–Laboratório Associado, Escola Superior de Biotecnologia, Universidade Católica Portuguesa, Rua Diogo Botelho 1327, 4169–005 Porto, Portugal; dmagalhaes@ucp.pt (D.M.); civrodrigues@ucp.pt (C.V.R.); sdsousa@ucp.pt (S.S.); jrcosta@ucp.pt (J.R.C.); pcteixeira@ucp.pt (P.T.)

**Keywords:** biopolymer functionality, citrus waste, polysaccharide characterisation, hot acid-assisted extraction, drying methods

## Abstract

Pectin is a naturally occurring biopolymer extensively used for applications in the pharmaceutical, biotechnology, and food industries, and is abundantly present in lemon by-products. Although lemon peels represent a highly promising raw material, the structure of pectin is strongly influenced by extraction and drying processes, and the resulting attributes remain insufficiently understood. The present study systematically investigates the impact of conventional hot acid extraction using three different acidifying agents (citric, sulphuric, and hydrochloric) in combination with two drying techniques (oven-drying and freeze-drying) on the physicochemical, structural, thermal, and viscosity–shear rate properties of pectin obtained from lemon by-products (*Citrus limon*, Portuguese Eureka variety) following the prior recovery of bioactive compounds (essential oils and phenolic compounds). The results demonstrated that citric acid extraction followed by freeze-drying yielded the highest pectin recovery, at approximately 26.7%, highlighting the suitability of this approach for efficient by-product valorisation. Oven-dried pectins exhibited higher moisture contents (8.5–10%) and lower lightness values (L* = 57.02–65.67), indicating darker colouration compared to freeze-dried pectins (L* = 78.82–83.75). All extracted pectins presented a degree of esterification (DE ≥ 50%), classifying them as high-methoxyl pectins. The galacturonic acid (GalA) content ranged from 37.6 to 48.9% for oven-dried samples and increased to 44.1–58.6% for freeze-dried samples. Furthermore, pectin obtained from lemon by-products exhibited a well-defined structural organisation and enhanced thermal stability, especially for freeze-dried pectin samples, and suitable rheological properties, with no statistically significant variations observed between different acids or drying conditions, supporting its technological suitability for applications in the food, cosmetic, and pharmaceutical industries.

## 1. Introduction

**Figure 1:**
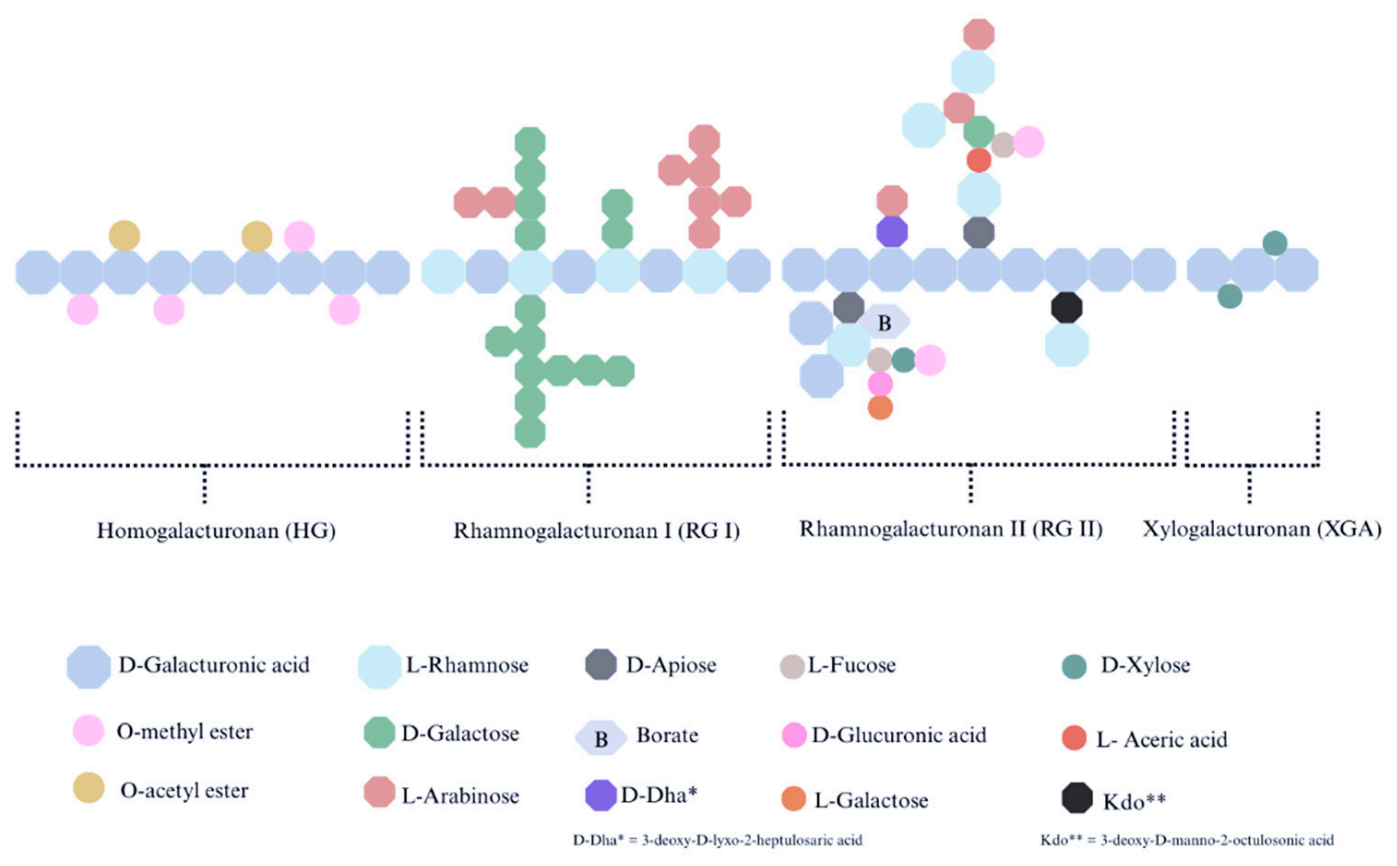
Pectin chemical structure and major structural domains, adapted from [1].

Pectin constitutes a family of complex polysaccharides that play an essential role in plant growth and development, primarily as a key structural component of the primary cell wall and middle lamella, contributing to mechanical resistance and protection against environmental challenges [1]. Pectin is mainly obtained from botanical sources, including citrus peels, apple pomace, and other fruit by-products, which are typically generated in large quantities by the agrofood industry [2,3,4]. In plants, pectin provides intercellular adhesion, structural stability, and mechanical strength, thereby supporting cell integrity and tissue cohesion, which are vital for survival in harsh environments characterised by extreme temperatures, pollutants, and other environmental stressors [5]. Pectin is a heteropolysaccharide composed of galacturonic acid and neutral sugars, with carboxyl groups that may be partially esterified with methyl and/or acetyl groups. Pectin is also recognised as one of the safest food additives due to its biocompatibility and non-toxic nature. Structurally, pectin consists of D-galacturonic acid units linked by α-(1,4) bonds, forming a linear chain interspersed with highly branched regions. Its complex structure includes several subdomains, including rhamnogalacturonan I (RG I), II (RG II), and xylogalacturonan (XGA), which are covalently linked to the homogalacturonan (HG) skeleton, the predominant domain of the pectin’s macromolecule (Figure 1) [6].

The degree of esterification (DE) is defined as the proportion of esterified carboxyl groups relative to the total number of galacturonic acid (GalA) units in the HG backbone and is a critical determinant of pectin functionality, particularly its gelling behaviour and other functional properties [7]. The degree of methylation (DM) refers specifically to the fraction of methyl-esterified carboxyl groups among the total number of GalA units and is commonly assessed using infrared spectroscopy. There are two main types of pectin: high methoxyl (HM, DE ≥ 50%) or low methoxyl (LM, DE < 50%). HM pectin forms gels at high soluble solid concentrations, particularly at acidic pH levels (typically 3.5), where gelation is driven by hydrogen bonding and hydrophobic interactions between methyl ester groups.

In contrast, LM pectin forms gels across a broader pH range (2.0–6.0) when calcium or other multivalent cations, such as Ca^2+^, are present through ionic crosslinking mechanisms commonly described by the “eggbox” model. HM pectin can be converted into LM by de-esterification, either chemically (alkaline treatment) or enzymatically (with pectin methylesterase) [8]. Despite sharing a common polysaccharide framework, pectin exhibits substantial structural and functional variability depending on its botanical origin and extraction process. These differences include molecular weight and distribution, neutral sugar side chains, esterification degree, ferulic acid content, proteins, and levels of methoxylation and acetylation, all of which strongly influence gel strength, viscosity, and application [9].

The structural complexity and variety of pectin make it an adaptable biomaterial. Due to the abundance of functional groups, pectin exhibits exceptional modification potential compared to other biopolymers. Additionally, it can undergo modifications through physical, chemical, and enzymatic processes. The abundance of functional groups in pectin confers diverse properties, and specific changes enable its use in multiple applications across food, pharmaceutical, and biomedical sectors [10]. For example, in the food industry, pectin is widely employed in edible films and coatings to preserve fresh produce and extend shelf-life [11,12,13,14], while also functioning as a gelling, thickening, stabilising, and emulsifying agent in products such as jams, jellies, dairy products and beverages. In the pharmaceutical and biomedical fields, pectin has also attracted considerable interest for controlled drug delivery systems, wound healing materials, tissue engineering scaffolds, and the encapsulation of bioactive compounds [5,15,16]. Its modifiable structure enables new applications, either independently or in combination with other bioactive compounds or biopolymers. Given its high added value and distinctive behaviour, pectin has attracted significant interest from both industry and academia.

Pectin characteristics, such as non-toxicity, emulsion properties, diverse chemical composition, biocompatibility, low production costs, availability, and high stability, reinforce its popularity as a biopolymer, making it appealing for industrial applications in advanced biomedical fields [17,18,19,20,21]. In addition, according to the U.S. Food and Drug Administration (FDA), pectin is listed as generally recognised as safe (GRAS) for use in food production for human consumption [22]. Therefore, it is important to extract pectin from reliable sources to obtain suitable polymers that meet the specific needs of the food industry and support sustainable raw-material utilisation [23].

Although pectin extraction from different types of citrus by-products has been widely investigated, there is still a lack of integrative studies correlating extraction acid chemistry and drying strategies with multiscale structure–function relationships, particularly regarding their combined effects on the molecular, structural, thermal, and rheological behaviour of pectin derived from lemon by-products.

The present study integrates multiple analytical techniques, including physicochemical property analysis, monosaccharide composition, molecular weight distribution, degree of esterification and methoxy content, thermal analysis (DSC), structural characterisation (FTIR, SEM, PXRD), and viscosity–shear rate profiles, to provide a comprehensive understanding of how different acid anions by comparing citric acid, hydrochloric acid (HCl), sulphuric acid (H_2_SO_4_), and pectin drying methods (oven-drying at 50 °C and freeze-drying) influence pectin properties. To the best of our knowledge, this study provides a multifaceted and systematic assessment that has not been extensively reported in pectin research, making our work particularly innovative. Additionally, a key feature of this study is the extraction of pectin from lemon by-products through an integrated valorisation process, wherein bioactive compounds, specifically essential oils, and phenolic-rich extracts, were previously extracted. This strategy aligns with circular economy and zero-waste principles, providing novel insights into the behaviour of pectin within a sequential biorefinery framework, rather than in its native form.

## 2. Materials and Methods

### 2.1. Reagents

Ethanol (96%) was purchased from LabChem (Santo Antão do Tojal, Portugal). Ultrapure water was obtained using a Milli-Q purification system (Merck, Darmstadt, Germany). Commercial citrus pectin, trifluoroacetic acid (TFA), sodium nitrite, sodium azide, sodium acetate, citric acid, sulphuric acid (H_2_SO_4_), sodium hydroxide (NaOH), and the monosaccharide standards L-fucose, D-(−)-arabinose, D-(+)-galactose, D-(+)-glucose, D-(+)-xylose, D-(+)-mannose, D-galacturonic acid (GalA), and D-glucuronic acid were purchased from Sigma-Aldrich (St. Louis, MO, USA). Hydrochloric acid (HCl) was purchased from Merck (Algés, Portugal).

### 2.2. Plant Material and Processing

The lemon by-products analysed were supplied by Frutas Tereso (Algarve, Portugal) in May 2023. They consisted of non-conforming lemons not suitable for consumer sale and therefore representative of industrial by-product streams. The lemon variety was *Citrus limon* Eureka (size 4/5), classified as category II (slight shape deviations, skin roughness, or healed mechanical marks, such as hail or rubbing scars, that do not compromise the inner flesh). Detailed information regarding agricultural practices, including pest management, fertilisation regimes, disease control measures, irrigation strategies, and soil characteristics, is provided in Magalhães et al. (2025) [24]. In addition, the lemon by-products (mainly peels) were ground using a Kenwood grinder (180 W; Moulinex, Alençon, France) for three minutes and then stored at −20 °C until pectin extraction.

### 2.3. Extraction of Lemon By-Product Pectin

**Figure 2:**
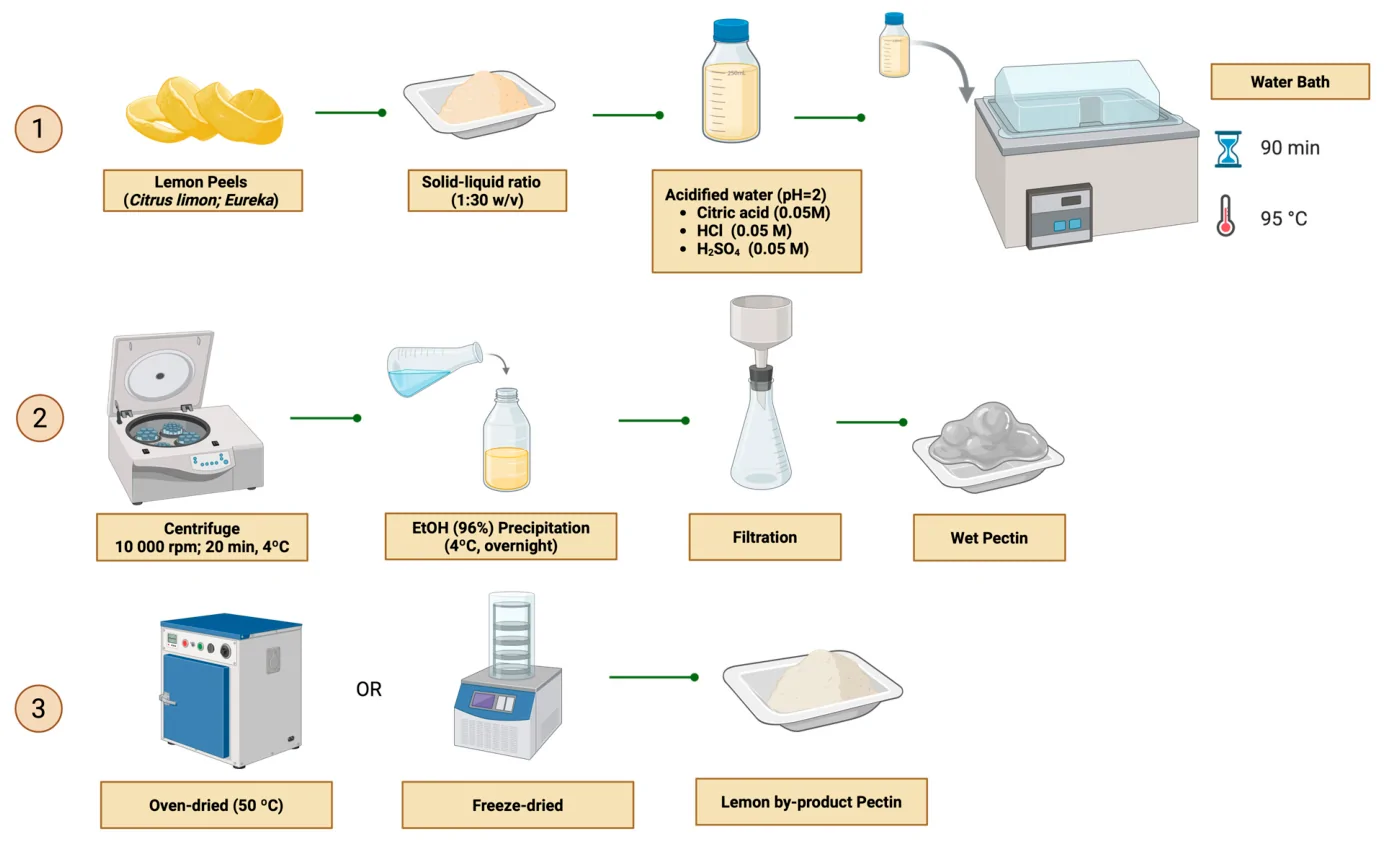
Pectin extraction workflow from lemon by-products, highlighting the comparison between acid type and drying method. Created in BioRender. Magalhaes, D. (2026) https://BioRender.com/thpz4ys.

Acidic extraction of lemon pectin was carried out according to the method described by Pasandide et al. (2018) [25] with some modifications to allow a comparative evaluation of acid type and drying strategy. The fresh peel (solid–liquid ratio of 1:30 (*w*/*v*)) was soaked in 0.05 M citric acid, 0.05 M hydrochloric acid (HCl), or 0.05 M sulphuric acid (H_2_SO_4_) aqueous solutions adjusted to pH 2, and then stirred. The solution was heated to 95 °C and maintained for 90 min in a water bath under stirring. After extraction, the mixture was centrifuged (10,000 rpm, 20 min, 4 °C) to eliminate impurities. Then, twice the volume of ethanol (96%) was added to the solution and stored at 4 °C overnight. Afterwards, pectin was filtered from the solution through gauze and dried. To evaluate potential differences in pectin structure and composition arising from post-extraction processing, two drying methods were applied. Therefore, after the extraction process, the pectin was either dried in an oven (INCU-LINE 150R Premium, VWR, Radnor, PA, USA) at 50 °C until constant weight was achieved or in a freeze-dryer (Armfield, UK) (Figure 2). The extraction of lemon by-product pectin was performed in three independent batches, each analysed in triplicate (*n* = 9).

The yield of extraction was calculated using the ratio between the dry weight of pectin obtained and the dry basis weight of lemon peels used for each extraction (Equation (1))

(1) $$Pectin\;Yield\;\left(\%\right)=\frac{Pectin\;dry\;weight\;(g)}{Dry\;basis\;weight\;of\;lemon\;peels\;(g)}\times 100$$

### 2.4. Pectin Characterisation

#### 2.4.1. Determination of Moisture, Ash and Protein

Moisture content, ash, and protein of lemon by-product pectin were determined in accordance with the Association of Official Analytical Chemists (AOAC) [26]. The moisture content was determined by drying pectin samples (2 g) in the oven at 105 °C for 24 h until a constant weight. Ash was determined by carbon removal using an initial 2 g of dry samples, which were heated for 5 h in a muffle furnace at approximately 550 °C. Furthermore, the protein content of pectin samples was determined by the Kjeldahl method, using a conversion factor of 6.25. The analysis was performed in triplicate for each batch.

#### 2.4.2. pH

The pH was measured for a 1% *w*/*w* solution of lemon by-product pectin using a pH metre (Crison micropH 2002, Barcelona, Spain) at room temperature. For each batch per sample, three replications were done.

#### 2.4.3. Colour

The colour of lemon by-product pectin was determined using a calibrated Chroma Metre CR-400 (Konica Minolta Sensing Inc, Osaka, Japan). It was expressed in terms of L* (lightness: 0 = black, 100 = white), a* (−a = greenness, +a = redness), and b* (−b = blueness, +b = yellowness). For each batch per sample, three replicates were performed.

#### 2.4.4. Monosaccharide Composition and D-Galacturonic Acid Level by HPAEC-PAD

The identification and quantification of neutral and acidic monosaccharides in the samples were performed by high-performance anion-exchange chromatography coupled with pulsed amperometric detection (HPAEC-PAD) on a Dionex ICS-5000 system (Dionex Corp., Sunnyvale, CA, USA). Samples were hydrolysed with 2 M trifluoroacetic acid (TFA) at 120 °C for 3 h, then dried in a speed vacuum. The recovered powder was then dissolved in ultrapure water and filtered (0.22 μm) before injection [27]. Separation was performed on a CarboPac PA10 (Thermo Fisher Scientific, Waltham, MA, USA) analytical column equipped with a guard column at a flow rate of 1.0 mL/min. The mobile phases consisted of ultrapure water (18.2 MΩ·cm, mobile phase A), 200 mmol/L NaOH (mobile phase B), and a mixture of 200 mmol/L NaOH with 1 mmol/L sodium acetate (mobile phase C). A multi-step gradient elution programme was applied to allow resolution of both neutral and acidic monosaccharides. The gradient programme was as follows: 88% A and 12% B (0–10 min); 92% A and 8% B (10–15 min); 88% A and 12% B (15–20 min); 50% A and 50% B (22 min); 50% A, 45% B, and 5% C (25 min); 50% A, 41% B, and 9% C (27 min); 50% A, 37% B, and 13% C (47 min); and 48% A and 52% C (50–55 min). After each run, the column was regenerated with 100% B for 10 min, followed by a 5 min re-equilibration at the initial conditions (88% A and 12% B). All eluents were purged and maintained under a nitrogen atmosphere to minimise carbonate contamination.

Quantification of monosaccharides was achieved using external standards. A set of external standards (fucose, arabinose, galactose, glucose, xylose, mannose, D-galacturonic acid, and D-glucuronic acid) with concentrations ranging from 200 to 1.562 mg/L was also prepared. Myo-inositol was added as an internal standard in both the standards and the samples to enable quantitative analysis of polysaccharide composition. In addition, to further elucidate the structure of lemon by-product pectin, the following proportions have been calculated: RG-I (%) = 2 Rha + Ara + Gal, HG (%) = GalA − Rha; HG (%) = GalA − Rha; R1 (%) = GalA/(Fuc + Rha + GluA + ArA + Gla + Xyl); R2 (%) = Rha/GalA; and R3 (%) = (Gal + Ara)/Rha.

#### 2.4.5. Molecular Weight (MW) Distribution by HPLC-SEC

The molecular weight (MW) of pectin from lemon by-products was measured using size exclusion chromatography (SEC) coupled with a chromatographic system, specifically a Beckman and Coulter 168 series HPLC system with a refractive index (RI) detector (Knauer, Berlin, Germany), as described by Campos et al. (2020) [28] with slight modifications to optimise pectin solubilisation and separation. Molecular separation was carried out using columns packed with hydroxylated polymethacrylate-based gel from Waters Ultrahydrogel SEC Columns (Waters, Milford, MA, USA) serially coupled as follows: UltraHydrogel Guard column (6 × 40 mm), 120 A, 250 A and 2000 À (7.8 × 300 nm). Dispersions of 5 mg/mL of pectin were prepared in ultrapure water (Milli-Q, Merck, Darmstadt, Germany) and allowed to hydrate overnight at room temperature (20–25 °C).

Afterwards, samples were filtered through a 0.22 μm nylon membrane and transferred to glass vials. 30 μL of sample was injected into the HPLC-SEC system, eluted with a mobile phase (previously degassed and filtered) composed of sodium nitrite (NaNO_2_; 0.1 M) and sodium azide (NaN_3_; 0.2 g/L) (Sigma-Aldrich (St. Louis, MO, USA)) in ultrapure water under an isocratic gradient at a continuous flow of 0.5 mL/min for 90 min at room temperature. Data acquisition and analysis were performed in triplicate for each batch using Karat32 5.0 software. The average MW of the displayed peaks were analysed and quantified by comparison to the retention times using a calibration curve (y= −0.1552x + 8.6878; R^2^ > 0.95) constructed with Shodex Pullulan P-82 standards (Showa Denko K. K., Tokyo, Japan): P-5 (6.1 kDa), P-10 (9.6 kDa), P-20 (21 kDa), P-50 (47 kDa), P-100 (107 kDa), P-200 (194 kDa), P-400 (337 kDa) to P-800 (642 kDa).

#### 2.4.6. Degree of Esterification (DE) and Degree of Methylation (DM)

The degree of esterification (DE) of the lemon pectin samples was determined by titration according to the method of Panwar et al. (2023) [29]. In short, 100 mg of each sample was dissolved in 2 mL of 96% (*v*/*v*) ethanol and 20 mL of dH_2_O. Afterwards, five drops of phenolphthalein were added to each pectin solution, which was then titrated with 0.1 M NaOH until the solution appeared a pale pink colour (V1). To the titrated solutions, 10 mL of 0.1 M NaOH was added, and the mixture was stirred for 15 min. Subsequently, 10 mL of 0.1 N HCl was added to each solution, and the mixture was stirred until the pink colour had completely disappeared. The samples were again titrated with 0.1 N NaOH until the pale pink colour reappeared (V2). The DE of each pectin sample was calculated using Equation (2).

(2) $${\mathrm{DE}}_{\mathrm{tritation}}\;(\%)=\frac{V2\;(\mathrm{mL})}{V1\;\left(\mathrm{mL}\right)+V2(\mathrm{mL})}\times 100$$

V1: Volume (mL) of 0.1 N NaOH used for the first sample titration, and V2: volume (mL) of 0.1 N NaOH used for the second sample titration.

In addition, the degree of methylation (DM) of pectin molecules reflects the degree of methylation of galacturonic acid units, which contributes to the overall degree of esterification. In the present study, to estimate DM, the conversion proposed by Han et al. (2024) [30] was adopted, assuming a theoretical maximum methoxy content of 16.3%, corresponding to 100% esterification. The conversion relationship between DM and DE was calculated using Equation (3).

*DM* (%) = *DE* × 0.163 (3)

#### 2.4.7. Fourier-Transform Infrared (FTIR) Spectroscopy Analysis

The dried lemon by-product pectin samples and commercial citrus pectin were analysed by FTIR spectroscopy. Infrared spectra were obtained by analysing the samples with a Spectrum 100 FTIR Spectrometer (Perkin Elmer, Waltham, MA, USA) using a total attenuated internal reflection (ATR) attachment in the spectral region between 500 cm^−1^ and 4000 cm^−1^. The resolution used was 2.0 cm^−1^, and each spectrum was produced by an average of 100 scans in transmittance (%T) mode.

In addition, the degree of esterification (DE) of pectin was also determined from FTIR spectra. DE is calculated based on the absorption bands at 1600–1630 cm^−1^ and 1740 cm^−1^, which correspond to the antisymmetric COO− and the C=O ester stretching vibration, using Equation (4) reported by Manrique and Lajolo (2002) [31].

DE_FTIR_ (%) = 124.7R + 2.2013  R = A_1740_/(A_1740_ + A_1630_) (4)

where A_1740_: the peak area corresponding to the esterified galacturonic acid (ca. 1740 cm^−1^) and A_1630_: the peak area corresponding to the free galacturonic acid (ca. 1630 cm^−1^).

#### 2.4.8. Scanning Electron Microscope (SEM) Analysis

The morphology of the pectin samples was evaluated using a Phenom XL G2 (Thermo Fisher Scientific, Waltham, MA, USA) scanning electron microscope (SEM). Sample powder was placed on top of the observation pins (covered with double-sided adhesive carbon tape (NEM tape; Nisshin, Nisshin-shi, Japan), and then coated with gold/palladium using a sputter coater (Polaron, Bretzfeld, Germany). Observations were performed with an acceleration voltage of 5 kV in high vacuum. The micrographs presented are representative of the overall powder morphology.

#### 2.4.9. Differential Scanning Calorimetry (DSC)

The DSC measurements were conducted in a nitrogen atmosphere (100 mL/min) using DSC 204 F1 Phoenix equipment (Netzsch, Waldkraiburg, Germany), which was calibrated with an indium standard. The samples, ranging from 3 to 5 mg, were placed in aluminium DSC pans and heated from 30 to 300 °C at 10 °C/min. All analyses were conducted in triplicate.

#### 2.4.10. Powder X-Ray Diffraction (PXRD)

Pectin obtained from lemon by-products was subjected to powder X-ray diffraction (PXRD) analysis using a Rigaku MiniFlex 600 diffractometer with Cu k*α* radiation, with a voltage of 40 kV and a current of 15 mA (3° ≤ 2θ ≥ 60°; step of 0.01 and a speed rate of 3.0 °/min). The relative crystallinity (RC) was calculated using Equation (5), where C_a_ is the crystallinity area, and A_a_ represents the amorphous area. All analyses were conducted in triplicate.

(5) $$\mathrm{RC}\;(\%)=(\frac{C_{a}}{C_{a}+A_{a}})\times 100$$

#### 2.4.11. Rheological Properties (Viscosity–Shear Rate Profiles)

Viscosity–shear rate profiles were performed to evaluate the physical properties of the lemon by-product pectin. A rotational rheometer CS-50 (Bohlin Instruments, Cranbury, NJ, USA), equipped with a cone and plate geometry (40 mm diameter, 4° angle), was utilised with a 0.15 mm gap, at 25 °C. The 1% (*w*/*v*) pectin samples were dissolved in distilled water, gently stirred to ensure homogeneity, and then transferred to the measurement plate. The viscosity (Pa·s) was measured over a shear rate range of 1–100 s^−1^. Measurements were performed in triplicate.

### 2.5. Statistical Analysis

Conventional statistical methods were used to calculate means and standard deviations of the three replicates per batch. Data were analysed using two-way analysis of variance (ANOVA), and if statistically significant differences were found, a Tukey post hoc test was performed at the 5% significance level (*p* < 0.05). The statistical analyses were conducted using GraphPad Prism Software (version 8).

## 3. Results and Discussion

### 3.1. Pectin Recovery Yield (%)

**Figure 3:**
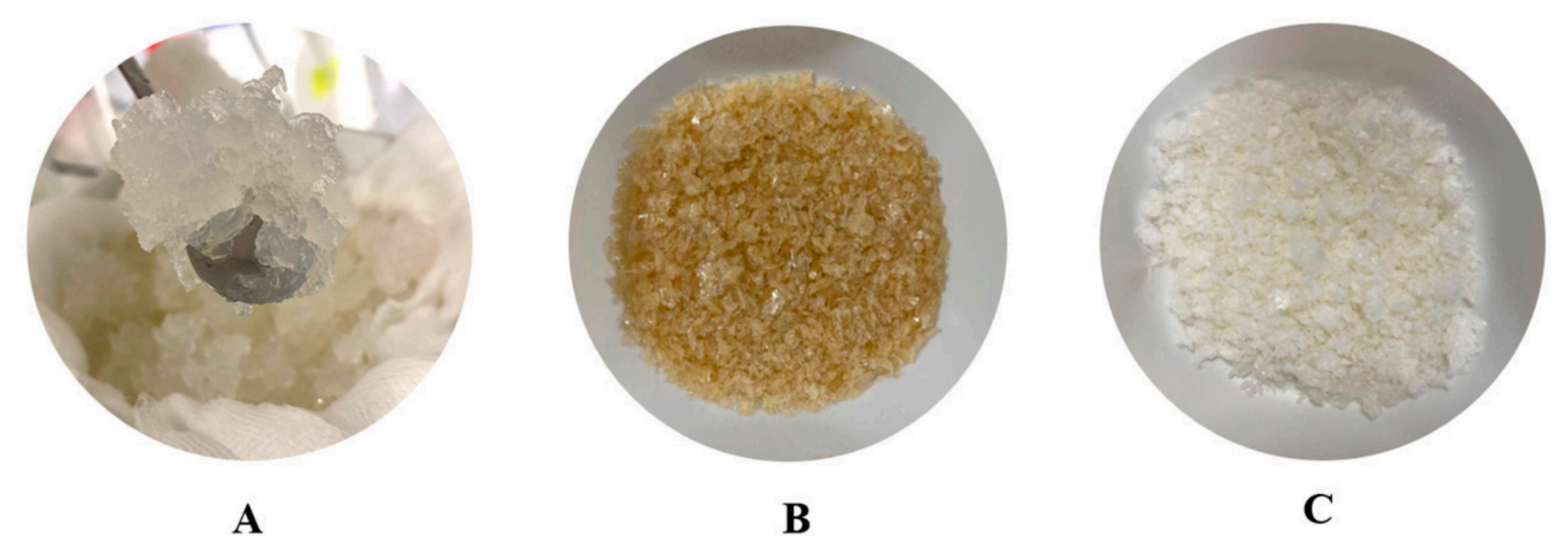
Visual aspect of lemon by-product pectin. (**A**) Wet lemon pectin. (**B**) Lemon pectin samples oven-dried at 50 °C after extraction. (**C**) Lemon pectin samples freeze-dried after extraction.

Different extraction variables were tested to obtain lemon by-product pectin, including the type of acid used (citric, HCl, or H_2_SO_4_) and the drying method (oven-dried or freeze-dried), at fixed conditions of time (90 min), temperature (95 °C) and pH (2). This experimental design allowed the independent and combined evaluation of extraction chemistry and post-extraction processing on pectin recovery efficiency. The visual appearance of lemon by-product pectin (wet, oven-dried, and freeze-dried) is shown in Figure 3.

The extraction yield for lemon by-product pectin ranged from 19.6% to 26.7%, based on the initial dry peel mass (Table 1). This yield range demonstrates an efficient recovery of pectin from lemon by-products under the experimental conditions applied. The freeze-drying process showed slightly higher pectin yields, suggesting reduced material losses during solvent evaporation, but the difference was not statistically significant (*p* > 0.05). The highest yield was achieved with citric acid, followed by H_2_SO_4_ and HCl. This result suggests that citric acid may provide a milder extraction environment, promoting pectin solubilisation while limiting acid-induced depolymerisation.

Pectin yield can be affected by various factors, including pectin source, pH, temperature, extraction duration, and the type of acid used, as well as post-extraction processing conditions. Our extraction yield is higher than a recent study conducted by Dambuza et al. (2024), who extracted pectin from lemon peels with HCl and reported maximum extraction yields ranging from 12.2% to 13.1%, which are significantly lower than the yields obtained in the present study [32]. These differences may be attributed to variations in extraction severity, raw material characteristics, or downstream processing conditions. Another study by Akhter et al. (2024) reported that the pectin yield from orange peels ranged from 6.20 to 21.63% [21]. The highest yield, 21.63 ± 0.25%, was achieved using sulphuric acid for 90 min. The trend in this article for pectin yield with different acids was H_2_SO_4_ > citric acid > HNO_3_ > HCl. Similarly, Kamal et al. (2021) reported a maximum yield of 22.45% from orange peel powder after 90 min, supporting our findings [20]. In addition, an optimisation study using a Box–Behnken experimental design reported by Panwar et al. (2024) was adopted to recover pectin from *Citrus limetta* peels. The maximum pectin yield (22.03 ± 0.13%) was achieved at 90 °C, 95 min, pH 1.8, and a solid-to-liquid ratio of 1:30 (*w*/*v*) [33].

These conditions and yields are highly comparable to those obtained in the present study using lemon by-products, indicating the robustness of our extraction methodology and its successful application for pectin recovery. Overall, the high yields obtained further highlight the suitability of lemon by-products as a valuable raw material for pectin extraction within a sustainable valorisation framework.

### 3.2. Proximate Composition

The moisture, ash, and protein content of lemon by-product pectin are shown in Table 1. The moisture content of oven-dried samples ranged from 8.52 to 10.00%, whereas substantially lower values were observed in the freeze-dried samples (1.88–2.47%). These results suggest that the drying method significantly affected the samples’ proximate composition (*p* < 0.05). As expected, freeze-drying resulted in notably lower moisture content than oven drying, reflecting its greater efficiency in removing water through sublimation under reduced pressure, which minimises structural collapse. This pronounced reduction highlights the strong influence of drying technique on residual water content. Our lemon by-product pectin has a moisture content similar to that of commercial citrus peel pectin (Sigma), which contains 10% moisture and is generally considered suitable for food-grade applications and long-term storage. Ash content ranged from 1.78 to 3.14% in oven-dried samples and from 0.14 to 0.32% in freeze-dried samples. The significantly lower ash content observed in freeze-dried samples may indicate a lower retention of inorganic salts during ethanol precipitation and drying processes [34,35]. However, the exact mechanism responsible for this difference cannot be confirmed based on the present results. The ash content increases as pectin yield decreases, suggesting that sugar content and other constituents increase substantially during fruit ripening [36]. Furthermore, ash content is commonly used as an indirect indicator of pectin purity and mineral residue associated with extraction conditions. From a food-grade perspective, high-quality pectin typically presents an ash content in the range of 1–10% [37]. The ash content obtained in this study falls within this range, suggesting an adequate purity of the extracted pectin and compliance with established quality standards for commercial pectins.

Furthermore, protein content ranged from 4.66 to 5.18% in oven-dried samples and from 4.99 to 6.07% in freeze-dried samples (*p* < 0.05), with a slight tendency toward higher values in freeze-dried samples, suggesting that freeze-drying may better preserve the protein fraction, likely due to the absence of elevated temperatures. Residual protein may originate from covalently or physically associated cell-wall proteins, which are commonly retained in citrus pectins and may influence interfacial and rheological properties.

### 3.3. pH and Colour Analysis

The pH values of the pectin samples are presented in Table 1. As expected, all pectin samples exhibited acidic pH values. Oven-dried pectin showed pH values ranging from 3.09 to 3.25, while freeze-dried pectin ranged from 3.06 to 3.29. These acidic values are characteristic of citrus-derived pectins and reflect residual carboxylic groups within the homogalacturonan backbone. Statistical analysis indicated that the drying method did not have a significant effect on the pH of the pectin samples extracted with citric acid (*p* > 0.05). These values are consistent with those reported by Ubieko et al. (2023), who reported a pH of 3.5 for pectin recovered from *Citrus sinensis* fruit peels [38].

The colour of pectin is a critical quality attribute, as it directly affects the visual appeal of the resulting solution or coating and, consequently, the overall appearance of the food product in which it is incorporated. For this reason, specific colour characteristics are often required by the food industry. The colour parameters (L*, a* and b*) of lemon by-product pectin are presented in Table 1. The pectin obtained in this study exhibited highly desirable colour characteristics, particularly in the freeze-dried samples, which showed a very light appearance, with L* values ranging from 78.82 to 83.75, a* values from 3.03 to 3.57, and b* values from 1.88 to 4.43. This enhanced lightness can be attributed to the low thermal load of freeze-drying, which minimises non-enzymatic browning reactions and pigment degradation. In contrast, pectin subjected to thermal dry treatment (oven-dried) displayed a slightly darker colour, as illustrated in Figure 3, with L* values between 57.02 and 65.67. This colour change is likely associated with thermal-induced browning reactions during prolonged exposure to elevated temperatures. Nevertheless, these samples maintained an appealing colour profile appropriate for food-industry uses, closely resembling the noted “light brown” shade of commercial citrus peel pectin (Sigma).

Similarly, Dambuza et al. (2025) reported L* values of 61.03 for lemon peel pectin and 69.96 for commercial pectin. The commercial pectin sample exhibited the highest lightness, which is favoured in industries that prefer lighter pectin for incorporation into clear or lightly coloured food systems [39].

### 3.4. Monosaccharide Composition and D-Galacturonic Acid Level

The monosaccharide composition analysis results are presented in Table 2, revealing D-galacturonic acid (GalA), glucose (Glc), arabinose (Ara), galactose (Gal) and rhamnose (Rha) as the predominant monosaccharides. These monosaccharides are characteristic of citrus-derived pectins and reflect the polysaccharide heterogeneity of cell wall extracts. GalA content in food plays a crucial role in shaping various chemical and sensory attributes of the matrix, including pH, total acidity, microbial stability, sweetness, and overall quality and acceptability. From a structural perspective, GalA is the major building block of pectin and directly influences its gelling and rheological behaviour. Furthermore, GalA serves as the structural foundation for the homogalacturonan and rhamnogalacturonan (RG) regions within the pectin structure. Meanwhile, Gal and Ara, rather than being indistinguishable isomers, serve as the primary neutral sugars in the RG-I and HG side chains, respectively. This distinction is important, as the abundance of Gal and Ara is commonly associated with increased branching and flexibility of the pectin macromolecule. The commercial citrus peel pectin (Sigma) exhibited a GalA content ≥ 74% (dry basis), which was significantly higher (*p* < 0.05) than that of the lemon by-product pectins (41–57%). This difference can primarily be attributed to the higher degree of purification and standardisation of commercial pectin, which is enriched in HG and depleted of neutral sugars, proteins, and inorganic components. In contrast, pectins extracted from lemon by-products are less refined and likely contain RG-I regions and other co-extracted cell wall polysaccharides, leading to a dilution of the relative GalA content.

To further elucidate the structure of lemon by-product pectin, the following proportions have been noted in the literature [30,40]. These empirical estimations are widely used to infer pectin domain distribution based on monosaccharide composition. According to this relationship, the proportions of HG and RG-I structures in lemon by-product pectin were higher with citric acid extraction, ranging from 47.43 to 57.07% and 8.76 to 10.82%, respectively, indicating that it is an HG-type pectin. This structural dominance of HG is desirable for food hydrocolloid applications, where gel formation and viscosity development are required. Additionally, the Rha-to-GalA ratio offers an indirect measure of the relative amounts of RG-I and HG in pectin. In the RG-I region, Rha residues usually connect to Gal and Ara side chains. The molar ratio of (Gal + Ara) to Rha helps assess the extent of branching within RG-I. For lemon by-product pectin extracted in citric acid, the ratio that demonstrated the best extraction and best preservation of the biopolymer’s molecular structure ranged from 3.84 to 5.21%, rather than being expressed as a percentage, suggesting the presence of Ara and Gal side chains, which enhance its structural and functional properties. Higher branching in RG-I regions has been associated with improved emulsifying capacity and water-holding behaviour in pectin systems.

A recent study performed by Li et al. (2024) evaluated citrus peels pectin extraction using organic acids, ultrasonication, and/or microwave-assisted approaches, demonstrating that the extraction strategy markedly influences pectin structural composition [41].

Across the evaluated methods, GalA content ranged from 44.17 to 70.88%, while neutral sugar fractions showed substantial variability, with arabinose + xylose between 6.42 and 31.81%, galactose between 8.33 and 20.67%, and rhamnose between 3.47 and 11.59%. Importantly, pectin obtained by citric acid extraction in the absence of ultrasonication or microwave assistance, under conditions closely aligned with those of the present study, displayed a monosaccharide profile consistent with partial preservation of branched rhamnogalacturonan domains. The reported composition consisted of 0.69 ± 0.05% mannose, 11.59 ± 0.16% rhamnose, 5.10 ± 0.14% glucose, 10.68 ± 0.37% galactose, 16.20 ± 0.20% arabinose + xylose, and 55.74 ± 0.21% galacturonic acid, corresponding to a Rha/GalA ratio of 0.21. The relatively high rhamnose and neutral sugar contents suggest substantial retention of RG-I domains, in contrast to the more GalA-enriched pectins obtained under intensified extraction conditions. These findings are consistent with our results and support the suitability of mild organic acid extraction for preserving pectin structural complexity relevant to hydrocolloid functionality. These observations align closely with the structural profiles obtained in the present work.

Wani and Uppaluri (2023) [42] reported that pectin extracted from pomelo fruit peel using ultrasound-assisted extraction and conventional hot acid extraction exhibited galacturonic acid contents of 65.5% and 52%, respectively. The comparatively lower galacturonic acid content observed in the conventional hot acid extraction pectin is likely due to the prolonged thermal exposure inherent to this method, which may promote partial hydrolysis or solubilisation of GalA-rich homogalacturonan domains. Furthermore, monosaccharide composition analysis reported by Qi et al. (2023) [18] indicated that pectin extracted from citrus physiological premature fruit drop is rich in the rhamnogalacturonan I (RG-I) domain (50.40%), with long arabinose and galactose side chains (32.02%), and a galacturonic acid content of 50.56%. Furthermore, a study conducted by Guo et al. (2024) [17] revealed that pectin extracted from citrus peels is composed of GalA, Ara, Gal, and Glc. Similar to our findings, GalA was the most abundant monosaccharide, comprising 61.98–85.34% of the total monosaccharide content. Moreover, these results suggest that, under high-temperature acid extraction conditions, partial degradation of the pectin main chain occurs, preserving most of the neutral sugar side chains. Taken together, these findings confirm that mild organic acid extraction, as applied in this study, favours the retention of structurally complex pectin domains that are relevant for hydrocolloid functionality and application performance.

### 3.5. Molecular Weight (MW) Distribution by HPLC-SEC

**Figure 4:**
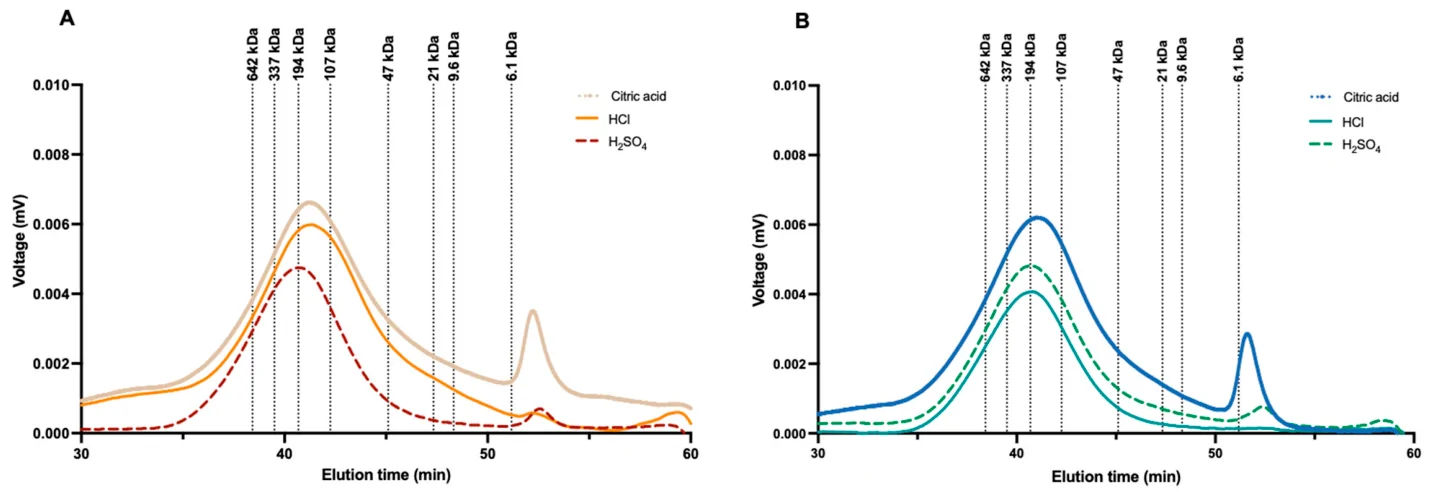
Molecular weight (MW) distribution of lemon by-product pectin determined by size exclusion chromatography (SEC) following acid-assisted extraction using citric acid, sulphuric acid (H_2_SO_4_), and hydrochloric acid (HCl). (**A**) Oven-dried pectin. (**B**) Freeze-dried pectin.

The average molecular weight (MW) plays an important role in defining the structure and functional behaviour of polysaccharides, particularly their gelling capacity. MW strongly influences viscosity, gel strength, and network formation in pectin-based systems. The chromatographic profiles of the lemon by-product pectin extracted with the three acids shown in Figure 4 indicate a broad MW distribution ranging from approximately <6.1 to 642 kDa, reflecting the heterogeneous nature of pectin macromolecules extracted under acidic conditions. Across all samples, the most prominent fraction eluted at around 194 kDa, indicating that this is the dominant molecular weight population in the extracted pectins and suggesting a balance between polymer integrity and partial depolymerisation. A quantitative analysis of the molecular weight of the pectin samples was performed using a calibration curve generated from molecular weight standards (Table 3). Based on the calculated molecular weight, the freeze-dried samples exhibited the highest molecular weights, ranging from 198.00 to 233.80 kDa, with the HCl-treated sample showing the highest MW. In comparison, the oven-dried samples presented slightly lower molecular weights, ranging from 184.02 to 226.40 kDa.

Comparison of drying methods within each acid treatment revealed broadly similar molecular weight profiles. However, some notable differences were observed. Citric acid extracted pectin, both oven-dried (Figure 4A) and freeze-dried (Figure 4B), exhibited a distinct and pronounced low-molecular-weight peak closer to 6.1 kDa, suggesting a partial depolymerisation during extraction or the presence of shorter pectic fragments co-precipitated during ethanol precipitation. The occurrence of these low-molecular-weight species could be minimised by incorporating a dialysis step against water, using membranes with an appropriately low molecular weight cut-off, allowing the removal of smaller pectic fragments prior to drying. Such post-extraction purification steps are commonly applied when tailored MW distributions are required for specific applications. In contrast, HCl-extracted pectin showed no evidence of low molecular weight fractions under either drying method. This indicates greater structural stability during both extraction and post-processing, with HCl causing minimal degradation of pectin chains under the conditions applied. Interestingly, this chemical stability is consistent with the smoother and more uniform surface morphology observed by SEM for the HCl pectins, suggesting a direct relationship between macromolecular integrity and microstructural organisation.

Muñoz-Almagro et al. (2018) [43] characterised industrial citrus pectin (pure) and commercial pectin-derived products containing maltodextrin or added sugars using two detectors (ELSD: evaporative light scattering detector and RID: refraction index detector). The pure citrus pectin exhibited a relatively narrow MW distribution, ranging from 317 to 434 kDa. In contrast, the pectin–maltodextrin product displayed a more complex profile, characterised by a high molecular weight peak (603–693 kDa) followed by three additional lower molecular weight fractions at approximately 20 kDa, 0.8–0.9 kDa, and 0.2 kDa. A similar pattern was observed for the pectin–sugar formulation, which also presented a dominant fraction at 480–492 kDa, followed by components at 36.4–38 kDa, 0.8–0.9 kDa, and 0.2 kDa. The presence of these low-MW fractions was attributed to added carrier agents and formulation components. Additional variability has been reported by Liu et al. (2022), who extracted pectin from ten citrus peel cultivars using the hot acid method and dialysed (molecular weight cut-off 3.5 kDa), obtaining MW values ranging from 336 to 985 kDa [44]. This wide range highlights the strong influence of cultivar and purification strategy on final MW values. These findings collectively demonstrate that the molecular weight of citrus pectin is strongly influenced by factors such as cultivar, extraction conditions, drying methods, and analytical detection techniques. The MW values obtained in the present study fall within the range reported for food-grade citrus pectins and are therefore suitable for hydrocolloid and food packaging applications.

### 3.6. FTIR Spectral Analysis

**Figure 5:**
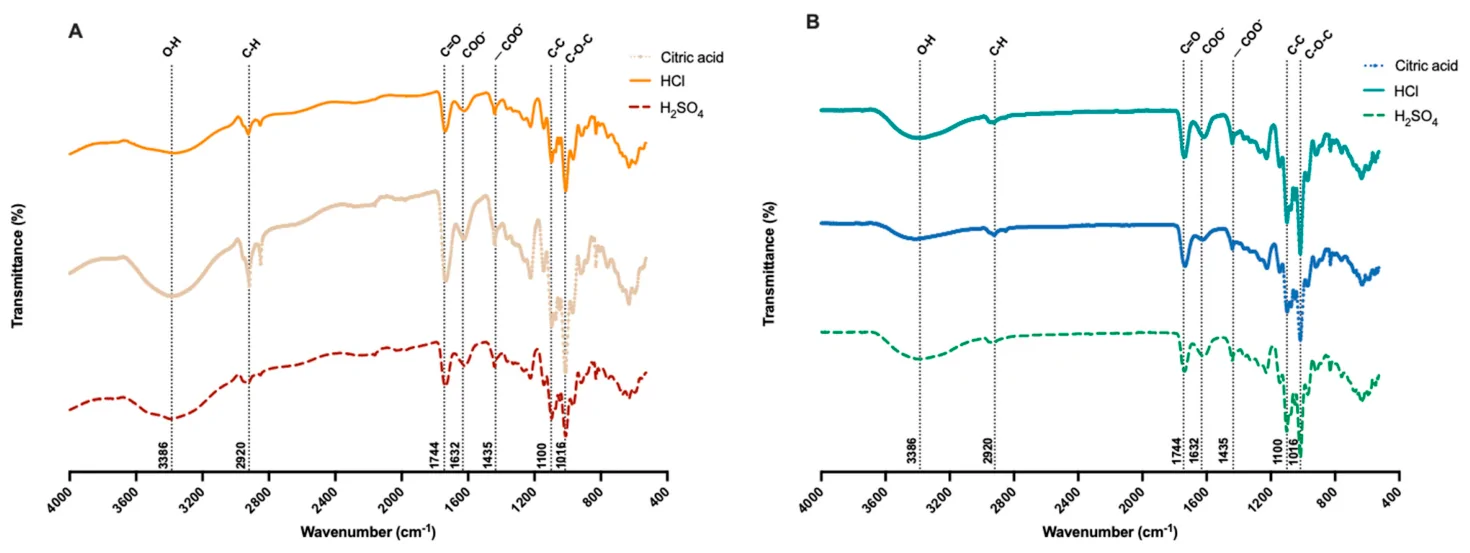
FTIR spectra of pectin extracted from fresh lemon peel under different extraction conditions. (**A**) Lemon by-product Pectin oven-dried at 50 °C after extraction. (**B**) Lemon by-product pectin freeze-dried after extraction; citric acid pectin extracted according to the conventional method using citric acid-acidified water; HCl–pectin extracted according to the conventional method using HCl-acidified water; H_2_SO_4_–pectin extracted according to the conventional method using H_2_SO_4_-acidified water.

Lemon pectin extracted using different acid treatments and oven (Figure 5A) or freeze-dried (Figure 5B) was analysed by ATR-FTIR spectroscopy, which is a widely used, non-destructive technique for assessing pectin chemical structure and esterification patterns. All samples exhibited spectra characteristic of pectin, confirming that the extraction processes preserved the major structural features of this polysaccharide. The presence of analogous chemical groups among the samples is consistent with previously reported results.

FTIR profiles for citrus pectin [30,41,45]. Furthermore, commercial citrus pectin was analysed and exhibited structural characteristics similar to those of lemon by-product pectin, supporting the comparability between the extracted samples and food-grade commercial pectins.

Across all spectra, the typical pectin absorption bands were observed. The absorption band at 1744 cm^−1^ is ascribed to the presence of the C=O bond in both the methyl ester group (COOCH_3_) and undissociated carboxylic acid (COOH). In contrast, the band occurring at 1632 cm^−1^ originates from the asymmetric stretching vibration of the carbonyl group in the carboxylate ion (COO−). Notably, the region from 1100 to 1016 cm^−1^ represents the carbohydrate fingerprint zone, which contains characteristic signatures specific to polysaccharides and reflects vibrations associated with C–O, C–C, and glycosidic linkages and is considered indicative of the polysaccharide backbone integrity.

Comparison of spectra according to the acid used for extraction indicates that, for both oven-dried pectin (Figure 5A) and freeze-dried pectin (Figure 5B), the typical pectin structure was preserved. No additional peaks associated with chemical degradation or contaminant functional groups were detected. However, the freeze-dried samples exhibited less intense and less well-defined O–H stretching bands in the 3700–3000 cm^−1^ region (*p* > 0.05). This reduction may be related to the greater moisture removal capacity of freeze-drying, which decreases hydrogen-bonded water molecules associated with hydroxyl groups in the pectin matrix. In addition, the band at approximately 2920 cm^−1^, attributed to aliphatic C–H stretching, appeared more pronounced in the oven-dried samples. This may be related to thermal effects during drying, which can enhance the relative intensity of aliphatic vibrations through molecular rearrangements or by partially concentrating waxy or lipid residues naturally present in lemon peel. Such thermal effects have previously been associated with subtle changes in the pectin microenvironment rather than backbone degradation. Overall, the ATR-FTIR analysis confirms that the extracted lemon by-product pectin retains the typical functional groups of pectin structure, and the type of acid used for extraction does not significantly disrupt its chemical integrity.

### 3.7. Degree of Esterification (DE) and Degree of Methylation (DM)

The degree of esterification (DE) and degree of methylation (DM) of lemon by-product pectin were evaluated across various extraction variables (three acids and two drying methods), through two methods: titration and FTIR spectroscopy, as shown in Table 4. Briefly, DE is defined as the percentage of esterified -COOH groups in pectin and can be classified as low-ester (DE < 50%) or high-ester (DE ≥ 50%) [7]. In addition to DE, the degree of methoxylation/methoxy content (DM) is another key indicator of pectin structure. DM represents the amount of methoxyl groups (–OCH_3_) attached to the galacturonic acid units, reflecting how extensively the polymer is methyl-esterified. Although DM and DE are related, since methyl esterification contributes to the total esterified groups, they describe different aspects of pectin modification. Therefore, both DE and DM play crucial roles in determining the functional properties of pectin across various applications, including gelling behaviour, setting rate, and calcium sensitivity [4].

As shown in Table 4, the lemon by-product pectins exhibited consistently high degrees of esterification (DE) across all extraction acids and drying methods, confirming that all samples fall within the category of high-ester pectins (DE ≥ 50%). DE was quantified using two complementary analytical techniques: classical titration and FTIR spectroscopy, which provided comparable but not identical results due to intrinsic methodological differences.

DE values determined by titration represent a direct quantitative measure of free and esterified carboxyl groups through neutralisation reactions. In contrast, FTIR spectroscopy estimates DE based on the relative intensities of characteristic ester carbonyl absorption bands. As expected, FTIR measurements were generally slightly higher than titration for most samples. This is likely due to spectral overlap in the ester carbonyl region, which can slightly overestimate ester functionalities [46]. FTIR analysis revealed no statistically significant differences (*p* > 0.05) among the samples obtained using different acids and drying techniques. Despite absolute value differences, both methods consistently identified the same esterification trends.

Among all treatments, the lowest DE was obtained with citric acid-extracted, oven-dried pectin (titration: 69.28%; FTIR spectroscopy: 76.33%), indicating that citric acid yields a less esterified polymer under these extraction conditions. In contrast, pectins extracted with mineral acids (HCl and H_2_SO_4_) consistently showed higher DE values, with HCl-extracted samples reaching the highest esterification levels (e.g., oven-dried: 88.39% by titration). This suggests that stronger acids may more effectively preserve methyl ester groups or reduce their hydrolysis during the extraction step, resulting in a more esterified pectin structure under controlled extraction times.

Drying also affected DE to some extent. Freeze-dried pectins generally showed slightly higher DE values than oven-dried pectins when measured by titration, especially for citric acid and H_2_SO_4_ extractions. As titration directly measures the functional groups, this difference suggests that thermal exposure during oven drying may promote partial demethylation. However, the FTIR spectroscopy did not show the same magnitude of variation between drying methods, indicating that FTIR spectroscopy is less sensitive to subtle changes in ester group hydrolysis. In addition, across all analytical conditions, extracted lemon by-product pectins remained within the high-methoxyl (HM) pectin range.

The findings of Akachat et al. (2025) are consistent with ours. They extracted pectin from lemon peel powder with three acids: HCl, nitric acid, and citric acid, and reported a methoxyl content of 8.07 ± 0.22% and a DE of 80.33 ± 1.52% [47]. The DE value is influenced by several factors, including the acid concentration (pH), extraction time, and temperature. For example, higher acid concentrations (low pH), longer extraction times, and elevated temperatures tend to promote de-esterification, thereby decreasing the DE value.

Based on the higher DE value obtained in this study, the pectin produced can be classified as HM pectin. Specifically, when the DE exceeds 72%, pectin is considered fast-setting and is commonly used in the food industry, including in the production of jams and jellies. Furthermore, in recent years, several studies have classified lemon pectin by-products as HM [29,48]. The consistency of DE and DM values across extraction acids and drying methods supports the technological reliability of lemon by-products as a source of high-quality HM pectin, suitable for industrial hydrocolloid applications.

### 3.8. Scanning Electron Microscopy (SEM) Analysis

**Figure 6:**
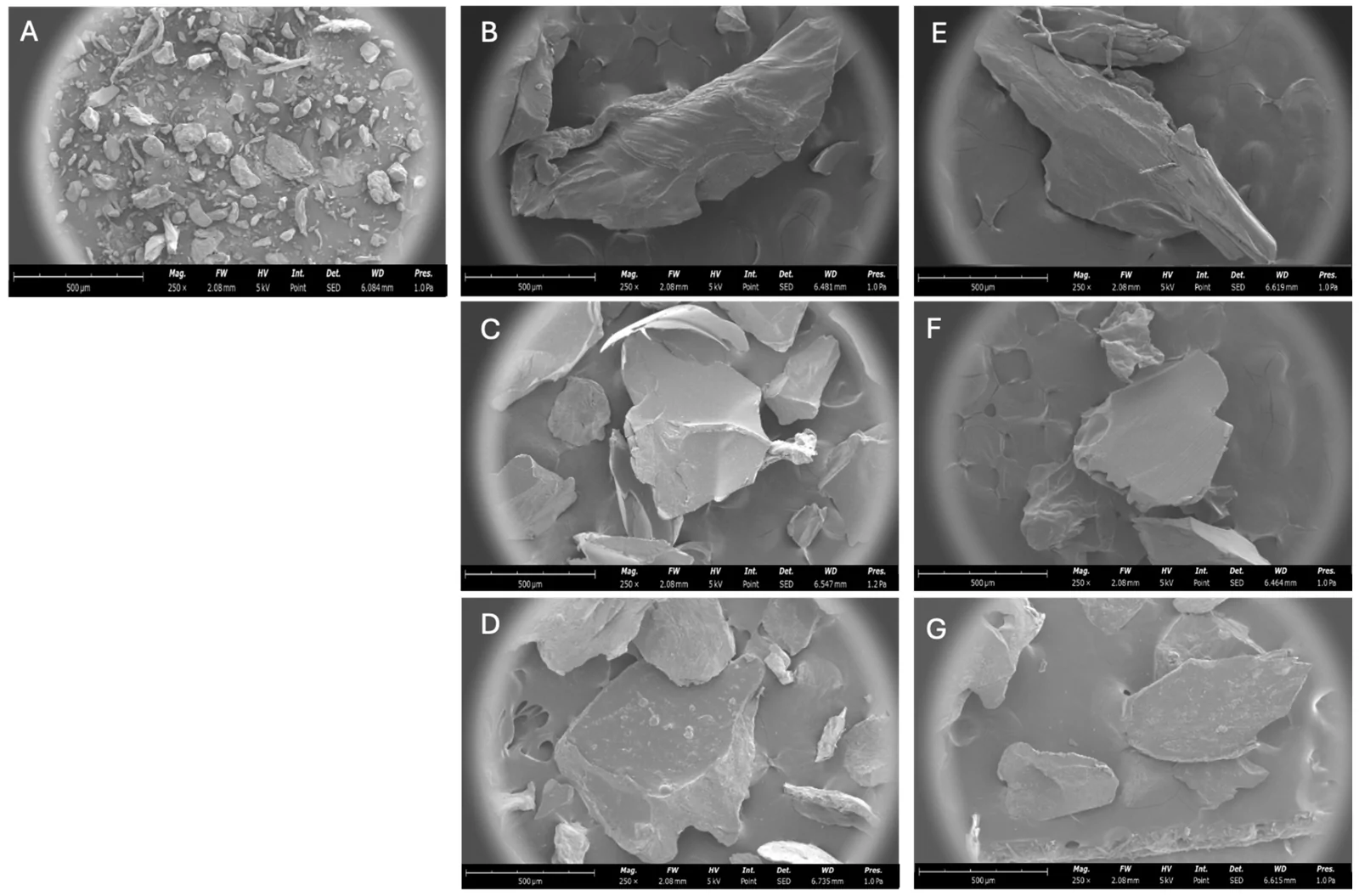
Scanning electron microscopy (SEM) micrographs (Scale bar: 500 μm) of the surface structure of commercial citrus pectin and lemon by-product pectin extracted using different acids and drying methods. (**A**) Commercial citrus pectin; (**B**–**D**) lemon by-product pectin oven-dried at 50 °C after extraction ((**B**): Citric acid; (**C**): HCl; (**D**): H_2_SO_4_); (**E**–**G**) lemon by-product pectin freeze-dried after extraction ((**E**): Citric acid; (**F**): HCl; (**G**): H_2_SO_4_).

The surface morphology of the commercial citrus pectin and the oven- and freeze-dried lemon by-product pectin obtained using different extraction acids was evaluated by SEM (Figure 6). The micrographs revealed that all pectin samples exhibited irregular, compact, flake-like structures with relatively smooth surfaces and no obvious macroporosity, consistent with the appearance of dried pectic polysaccharides [49]. This morphology is typical of pectins precipitated by alcohol and subsequently dried. Commercial citrus pectin (Figure 6A) exhibited a finer and more fragmented particulate structure, consistent with its powdered industrial form. The small particles presented smooth, uniform surfaces and relatively consistent size distribution. These features are generally associated with controlled industrial milling and post-processing operations. Such morphological features typically benefit dispersion in aqueous systems, promoting solubility and facilitating interactions with other food components. The increased surface area of smaller particles facilitates rapid hydration and binding [32]. In contrast, the extracted lemon by-product pectin samples (Figure 6B–G) appeared as larger, thicker flakes, reflecting the minimal grinding applied before SEM analysis.

This difference highlights the influence of post-extraction handling rather than intrinsic structural differences in the pectin polymer. Despite pronounced differences in particle size, the overall surface textures of the extracted pectins were generally comparable across samples, regardless of the extraction acid or drying method used. This suggests that neither acid type nor drying strategy induced major morphological disruption at the microscale. A subtle distinction was observed in the pectins extracted with HCl (Figure 6C,F), which exhibited slightly smoother, more uniform surfaces than those obtained with citric acid or H_2_SO_4_.

Although not sufficient to indicate significant structural alterations, this trend may suggest a minor effect of HCl on surface refinement during extraction.

Overall, the findings suggest that, under the tested conditions, the acid extraction and drying process did not markedly disrupt the intrinsic flake-like morphology of pectin, with only minor variations in surface smoothness observed between treatments. Such morphological stability is desirable for food-grade pectins, as it ensures predictable hydration and functional behaviour across processing conditions.

### 3.9. Differential Scanning Calorimetry (DSC) Analysis

**Figure 7:**
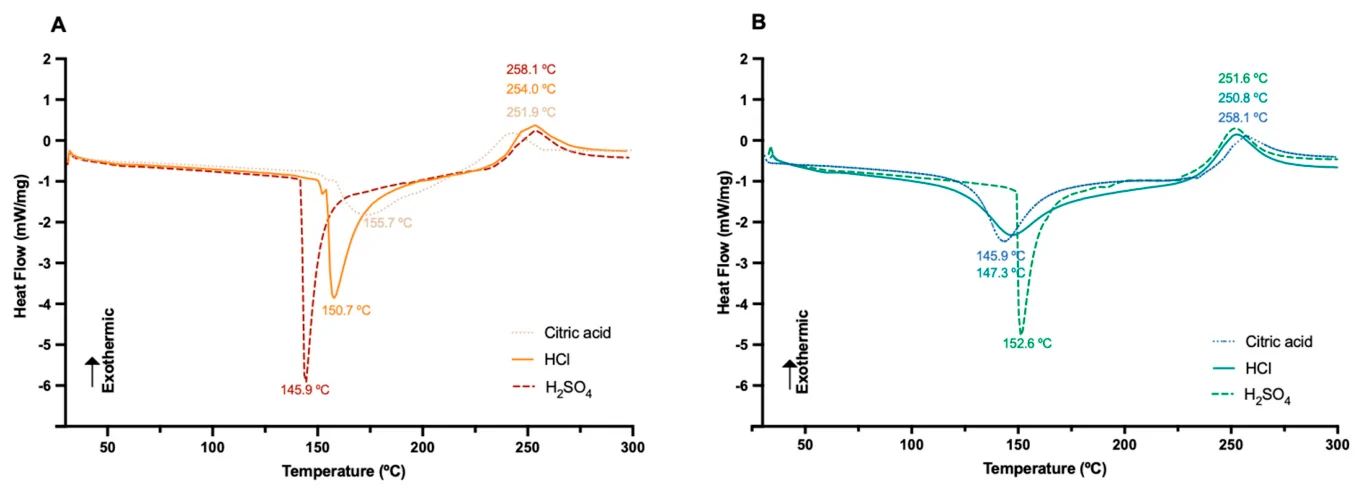
Differential scanning calorimetry (DSC) thermograms of lemon by-product pectin following acid-assisted extraction with citric acid, hydrochloric acid (HCl) and sulphuric acid (H_2_SO_4_). (**A**) Oven-dried lemon pectin. (**B**) Freeze-dried lemon pectin.

Figure 7 presents the differential scanning calorimetry (DSC) curves of lemon by-product pectin obtained using different drying techniques (A: pectin oven-dried at 50 °C; B: freeze-dried pectin). DSC analysis provides insight into thermal transitions associated with water binding, molecular organisation, and thermal stability of pectin polymers. As expected, the two thermograms exhibit distinct thermal behaviours, reflecting the influence of the drying method on the structural and thermal properties of the resulting pectin. For the oven-dried samples (Figure 7A), an initial endothermic peak was observed between 145.9 °C and 155.7 °C, with citric acid-extracted pectin exhibiting the highest melting temperature (155.7 °C) (*p* > 0.05).

For freeze-dried pectin (Figure 7B), melting temperatures ranged from 145.9 °C to 152.6 °C (*p* > 0.05). These endothermic transitions are commonly attributed to the evaporation of strongly bound water and to the disruption of hydrogen-bonded networks within the pectin matrix, providing valuable information on pectin’s hydration characteristics and internal organisation. Such transitions are often linked to pectin’s gel-forming and water-holding capacity. A second exothermic peak, associated with thermal degradation, appeared in all oven-dried samples within a narrow temperature range (251.9–258.1 °C), with citric-acid pectin degrading slightly earlier (251.9 °C). Freeze-dried samples showed similar behaviour, with exothermic peaks between 251.6 °C and 258.1 °C. However, despite the similar thermal degradation behaviour, significant differences between the drying techniques (*p* < 0.05) were observed for samples treated with citric and sulphuric acids. This exothermic peak corresponds to the onset of polysaccharide backbone degradation, including decarboxylation and depolymerisation reactions. Similar to the oven-dried samples, sulphuric acid-extracted pectin again exhibited the most pronounced melting transition. In both drying methods, sulphuric acid extraction produced pectin with the most intense endothermic melting peak, suggesting a more defined or crystalline thermal domain. This behaviour may reflect stronger intermolecular interactions or partial molecular ordering within the polymer network. Table 5 summarises the DSC-derived thermal parameters of pectin samples. Freeze-dried pectin showed the highest melting enthalpy (ΔH_m_), particularly for citric acid extracted samples (ΔH_m_ = 256.1 J/g), followed by commercial citrus pectin (ΔH_m_ = 239.1 J/g). Higher ΔH_m_ values indicate stronger intermolecular interactions and greater structural stability, demanding more energy to transition from the solid to the liquid state. This enhanced thermal stability is often associated with preserved polymer integrity and reduced thermal history effects. Commercial pectin also displayed the highest degradation enthalpy (ΔH_d_ = 190.7 J/g), followed by HCl acid in freeze-dried pectin (ΔH_d_ = 159.1 J/g), indicating a more robust structure that requires more energy input to decompose. This observation is consistent with the smoother and more homogenous morphology observed by SEM (Figure 6F), suggesting coherence between thermal and microstructural properties.

The present research aligns with recent studies evaluating the thermal properties of citrus pectin. Yu et al. (2024) reported that the maximum exothermic peaks were between 242.9 °C and 260.1 °C for pomelo peel pectin. However, their samples exhibited no endothermic melting peaks, suggesting the absence of bound water [50]. In contrast, another study observed an initial endothermic transition between 72.03 and 94.85 °C and a degradation peak between 249 °C and 255.5 °C in lemon peel pectin. Their study also reported a markedly higher melting enthalpy for commercial pectin (ΔH_m_ = 400.2 J/g), indicative of strong intermolecular interactions and higher thermal stability [39]. Moreover, lemon by-product pectin exhibited good thermal stability, with freeze-dried samples generally demonstrating improved resistance to thermal transitions. These differences further highlight the strong influence of extraction, purification, and drying history on pectin thermal behaviour.

### 3.10. Powder X-Ray Diffraction (PXRD) Analysis

**Figure 8:**
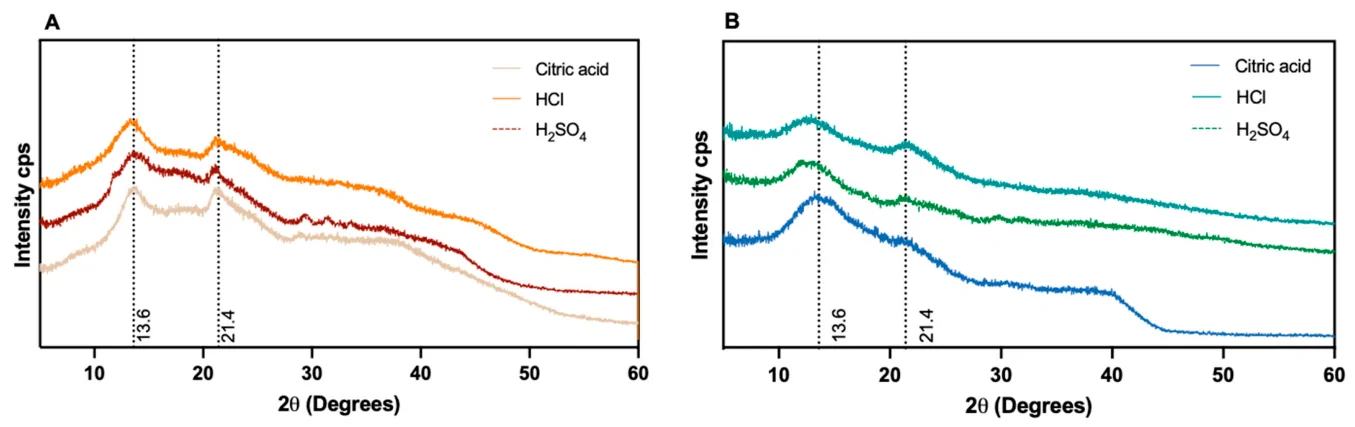
Powder X-ray diffraction (PXRD) patterns of lemon by-product pectin following acid-assisted extraction with citric acid, hydrochloric acid (HCl) and sulphuric acid (H_2_SO_4_). (**A**) Oven-dried lemon pectin. (**B**) Freeze-dried lemon pecti.

The powder X-ray diffraction (PXRD) patterns of the lemon by-product pectin (oven-dried at 50 °C and freeze-dried) using three different acids (Citric acid, HCl, H_2_SO_4_) are depicted in Figure 8. PXRD analysis provides information on the long-range molecular order and crystalline–amorphous balance of polysaccharide systems. All diffractograms showed two broad reflections centred at approximately 13.6° and 21.4°, characteristic of the semi-crystalline structure typically reported for pectin. The broadness of these peaks confirms the predominance of amorphous regions. A clear difference in diffraction patterns was observed between the two drying methods.

Oven-dried samples presented two sharp diffraction peaks, while freeze-dried samples showed only one, suggesting a slightly higher degree of molecular ordering induced by thermal drying. Thermal exposure during oven drying may promote limited chain rearrangement and packing, thereby increasing short-range order. Furthermore, the crystallinity of commercial citrus pectin analysed under the same conditions was 29.9%. The higher crystallinity in commercial and highly purified pectin has been associated with a high concentration of galacturonic acid residues and carboxyl groups, which enhance intermolecular interactions and promote ordered crystalline domains [51]. The lower crystallinity observed in lemon by-product pectin in this study may therefore be related to structural modifications induced during hot acid extraction, resulting in reduced chain organisation compared to commercial pectin. In particular, the presence of rhamnogalacturonan regions and residual neutral sugars may disrupt close chain packing. As a result, the pectin extracted in this study exhibits a more disordered structure compared to commercial pectin. The findings are consistent with the previous literature. Piacenza et al. (2022) reported crystallinity values of 37% for commercial pectin, 24% for lemon pectin, and 22% for grapefruit pectin, reinforcing the semi-crystalline character of citrus-derived pectins [52]. Similarly, Karim et al. (2022) described XRD patterns of lemon pectin showing characteristic reflections at 14.37°, 20.12°, and 32.20°, together with a broad amorphous halo between 15° and 25° (2θ) [45]. These features, including small crystalline contributions attributed to residual free sugars, closely align with the diffraction patterns obtained in the present study. The semi-crystalline behaviour observed here also parallels that reported for passion fruit pectin, which typically exhibits broad reflections near 13.8° and 21.1° [53].

Overall, the PXRD results confirm that lemon by-product pectin exhibits a predominantly amorphous structure with limited crystalline domains, a feature that is commonly associated with good solubility and functional flexibility in hydrocolloid applications.

### 3.11. Rheological Properties

**Figure 9:**
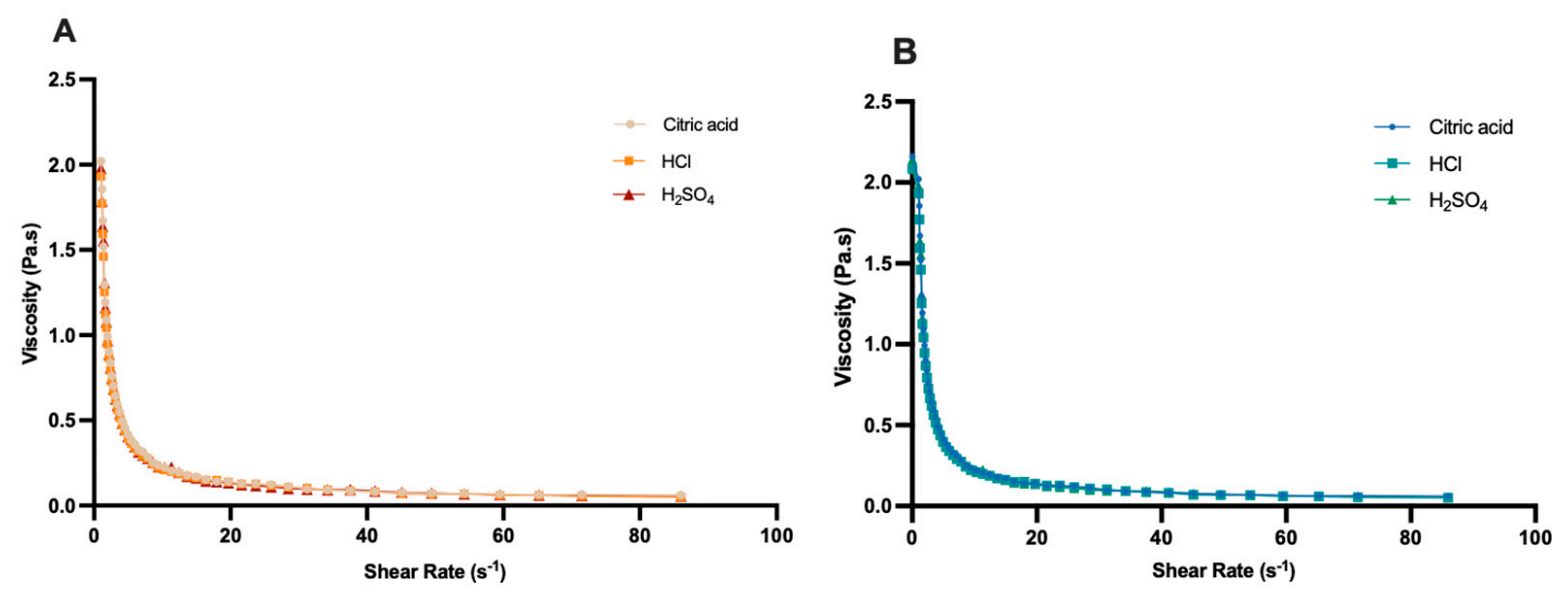
Viscosity–shear rate profiles of the lemon by-product pectin solutions (1%, *w*/*v*) (T = 25 °C). (**A**) Oven-dried lemon pectin. (**B**) Freeze-dried lemon pectin.

The flow behaviour of lemon by-product pectin samples (1%, *w*/*v*) is presented in Figure 9. The pectin solutions exhibited a progressive decrease in viscosity with increasing shear rate, indicating typical shear-thinning (pseudoplastic) behaviour. Such behaviour is characteristic of dilute to semi-dilute hydrocolloid solutions and is frequently reported for citrus-derived pectins. This rheological response is characteristic of polymer solutions, in which viscosity decreases with increasing shear rate due to the disentanglement and alignment of long molecular chains in the direction of flow. Statistical analysis showed that neither the type of acid used during pectin extraction nor the drying method (oven-drying or freeze-drying) had a significant effect on pectin solution viscosity (*p* > 0.05). This indicates that the fundamental flow behaviour at the tested concentration was not significantly altered by processing variables and is primarily governed by the intrinsic macromolecular structure of the pectin. Nevertheless, it should be noted that viscosity measurements alone do not fully describe the functional performance of pectin. Further studies evaluating parameters such as gel strength, gelation, solidification kinetics, and viscoelastic properties would provide a more comprehensive understanding of the relationship between the structural characteristics and technological functionality of the extracted pectins.

The shear-thinning behaviour can be attributed to the orientation of pectin macromolecules under shear. As the shear rate increases, the long pectin chains progressively align in the direction of flow, reducing intermolecular interactions and disrupting the transient network formed between adjacent chains. As a result, the pectin solutions approached Newtonian behaviour at high shear rates. This transition reflects the breakdown of weak physical entanglements and junction zones rather than permanent structural changes. Such rheological behaviour is closely related to the molecular structure of pectin, including chain length and degree of interaction [54]. Furthermore, the pseudoplastic region solutions become more pronounced at higher concentrations than at lower concentrations owing to the larger number of intermolecular networks formed between the pectin chains. At the 1% (*w*/*v*) concentration examined in this study, the solutions are likely near the overlap concentration, where chain interactions are sufficient to induce shear-dependent viscosity without forming a true gel network. Similar shear-thinning behaviour of pectin has been observed in various studies [50,55,56]. For example, a high-methoxy pomelo pectin (DE = 58%, MW = 353 kDa) exhibited Newtonian behaviour at concentrations below 0.6% (*w*/*v*) and pseudoplastic behaviour at concentrations above 0.6% (*w*/*v*) [57]. From a food application perspective, the pseudoplastic properties of pectin are highly advantageous, as they facilitate pumping and processing while imparting a desirable body and mouthfeel to food products. These properties make pectin an effective texturizer, stabiliser, and thickener in beverages, jams, dairy, and more, improving quality and ease of handling, and can be suitable for hydrocolloid applications [9].

## 4. Conclusions

This study investigated the effects of three different acids and two drying conditions on the physicochemical properties, structural characteristics, thermal properties, and rheological behaviour of pectin extracted from lemon by-products. By systematically combining acid-assisted extraction with different post-extraction drying strategies, this work provides an integrated evaluation of structure–property relationships in lemon by-product pectin. The main differences between the tested extraction acids and drying conditions were primarily attributable to the thermal, molecular, and surface properties of lemon-by-product pectin. Freeze-dried samples exhibited higher thermal stability, likely due to more efficient moisture removal and reduced hydrogen-bonding interactions, as indicated by DSC and FTIR analyses. This enhanced thermal behaviour reflects improved preservation of the polymer network during drying. SEM analysis revealed minor morphological differences, with HCl-extracted pectins exhibiting slightly smoother, more homogeneous surfaces, suggesting improved molecular integrity and reduced surface disruption during extraction. Regarding chemical functionality, freeze-dried pectins showed marginally higher degrees of esterification than oven-dried samples, suggesting partial demethylation induced by thermal drying, while citric acid extraction resulted in a distinct low-molecular-weight fraction, indicating a greater tendency for partial depolymerisation under organic acid conditions. The monosaccharide composition, particularly galacturonic acid (GalA) and the homogalacturonan region (HG), remains well preserved when using citric acid during freeze-drying pectins. This structural preservation is desirable for hydrocolloid performance and gel-forming capacity. In contrast, rheological behaviour remained unaffected by the extraction and drying conditions.

Furthermore, the bioactivities of the extracted pectin, namely antioxidant, antibacterial, and antidiabetic activities, were assessed; however, no significant activity was observed, and therefore these results were not included in the manuscript. This outcome is consistent with the chemical nature of pectin, which is not inherently bioactive in the absence of phenolic or low-molecular-weight compounds. This absence of bioactivity is likely associated with the prior removal of compounds with higher biological relevance, such as essential oils and phenolic constituents, during the initial extraction steps. Nonetheless, the lemon pectin obtained exhibited highly desirable physicochemical properties, including well-defined structural organisation, stable rheological behaviour, good thermal stability, a high degree of esterification (DE ≥ 50%), an elevated GalA content, and relatively high molecular weight. Together, these characteristics position lemon by-product pectin as a technologically competitive hydrocolloid comparable to commercial citrus pectins. These findings highlight that pectin derived from lemon by-products (*C. limon*, Portuguese Eureka variety) through the proposed cascade biorefinery approach exhibits physicochemical, functional and structural properties comparable to commercial citrus peel pectin, supporting the valorisation of lemon processing by-products within an integrated and sustainable biorefinery framework. Future studies will focus on exploiting this pectin as a biopolymerin combination with lemon by-product essential oils and/or phenolic compounds-rich extract to develop edible coatings that extend the shelf life of minimally processed fruits, thereby contributing to sustainable, clean-label food preservation strategies and the valorisation of citrus processing residues within a circular economy framework.

## Figures and Tables

**Table 1:** Extraction yield (%), chemical composition (moisture, ashes, protein %), pH, and colour analysis (L*, a*, b*) of lemon by-product pectin, according to different extraction variables.

Acid Conventional Extraction	Pectin Drying Method	Yield (%)	Moisture (%)	Ashes (%)	Protein (%)	pH	L*	a*	b*
Citric Acid	Oven-dried	23.83 ± 0.03 ^aA^	10.0 ± 0.74 ^aA^	2.14 ± 0.16 ^aA^	5.18 ± 0.07 ^aA^	3.09 ± 0.00 ^aA^	65.67 ± 3.56 ^aA^	6.41 ± 0.29 ^aA^	3.49 ± 0.27 ^aA^
	Freeze-dried	26.73 ± 0.02 ^aB^	1.88 ± 0.69 ^aB^	0.18 ± 0.08 ^aB^	6.07 ± 0.09 ^aB^	3.06 ± 0.05 ^aA^	78.82 ± 1.78 ^aB^	3.28 ± 0.12 ^abcB^	4.43 ± 0.33 ^aB^
HCl	Oven-dried	19.65 ± 0.01 ^bA^	8.52 ± 0.25 ^bA^	1.78 ± 0.07 ^bA^	4.68 ± 0.04 ^bA^	3.11 ± 0.02 ^aA^	57.02 ± 1.68 ^bA^	2.32 ± 0.07 ^bA^	3.03 ± 0.33 ^aA^
	Freeze-dried	22.67 ± 0.01 ^bB^	2.17 ± 0.90 ^bB^	0.14 ± 0.05 ^bB^	5.00 ± 0.12 ^bB^	3.29 ± 0.01 ^bB^	83.75 ± 0.99 ^bB^	3.03 ± 0.03 ^abB^	3.89 ± 0.58 ^aB^
H_2_SO_4_	Oven-dried	23.41 ± 0.00 ^cA^	8.84 ± 0.34 ^cA^	3.14 ± 0.21 ^cA^	4.66 ± 0.19 ^bA^	3.25 ± 0.02 ^bA^	60.55 ± 0.65 ^bA^	2.25 ± 0.11 ^bA^	2.83 ± 0.09 ^aA^
	Freeze-dried	25.38 ± 0.03 ^cB^	2.47 ± 0.79 ^cB^	0.32 ± 0.09 ^cB^	4.99 ± 0.07 ^bB^	3.19 ± 0.01 ^cB^	81.06 ± 0.48 ^abB^	3.57 ± 0.05 ^cB^	1.88 ± 0.27 ^bB^

Different lowercase letters (a–c) within the same column indicate significant differences among acids for the same drying method (*p* < 0.05). Different uppercase letters (A,B) within the same row indicate significant differences between drying methods for the same acid (*p* < 0.05), A. Oven-dried pectin. B. Freeze-dried pectin, according to Tukey’s multiple range test. Data are expressed as mean ± SD.

**Table 2:** Monosaccharide composition (%) of lemon by-product pectin.

			Monosaccharide Composition (%)						Molar Ratio (%)				
Acid Conventional Extraction	Pectin Drying Method	Rha	Ara	Gal	Glc	Man	Xyl	GalA	HG	RG-I	R1	R2	R3
Citric Acid	Oven-dried	1.50 ± 0.00 ^aA^	5.51 ± 1.04 ^aA^	2.30 ± 0.20 ^aA^	7.62 ± 0.70 ^aA^	0.19 ± 0.00 ^A^	0.11 ± 0.01 ^A^	48.93 ± 2.57 ^aA^	47.43	10.82	2.86	0.03	5.21
	Freeze-dried	1.50 ± 0.00 ^aA^	4.56 ± 0.00 ^aA^	1.19 ± 0.00 ^aB^	6.98 ± 0.00 ^aB^	0.20 ± 0.00 ^A^	0.12 ± 0.00 ^A^	58.57 ± 9.74 ^aB^	57.07	8.76	4.02	0.03	3.84
HCl	Oven-dried	3.19 ± 0.00 ^bA^	2.20 ± 0.00 ^bA^	0.96 ± 0.01 ^bA^	0.88 ± 0.00 ^bA^	Nd	Nd	45.28 ± 2.91 ^abA^	42.09	9.54	7.13	0.07	0.99
	Freeze-dried	3.19 ± 0.00 ^bA^	1.75 ± 0.64 ^bA^	0.62 ± 0.03 ^bB^	0.88 ± 0.00 ^bA^	Nd	Nd	44.48 ± 2.58 ^bA^	41.29	8.75	8.01	0.07	0.74
H_2_SO_4_	Oven-dried	Nd	1.72 ± 0.00 ^bA^	0.24± 0.00 ^cA^	0.88 ± 0.00 ^bA^	Nd	Nd	37.58 ± 1.87 ^bA^	37.58	1.96	19.15	-	-
	Freeze-dried	Nd	1.15 ± 0.00 ^bA^	0.24 ± 0.00 ^cA^	0.88 ± 0.00 ^bA^	Nd	Nd	44.14 ± 1.87 ^bA^	44.14	1.39	31.68	-	-

Rha: rhamnose; Ara: arabinose; Gal: galactose; Glc: glucose; Man: Mannose; Xyl: Xylose; GalA: galacturonic acid; HG: Homogalacturonan (HG = GalA − Rha); RG-I: Rhamnogalacturonan I (RG-I = 2Rha + Ara + Gal); R1: Linearity of pectin (R1 = GalA/(Fuc + Rha + GluA + Ara + Gla + Xyl); R2: Contribution of RG to pectin population (R2 = Rha/GalA); R3: Length of side chains attached to RG-I (R3 = (Gal + Ara)/Rha);. Nd: not detected. Different lowercase letters (a–c) within the same column indicate significant differences among acids for the same drying method (*p* < 0.05). Different uppercase letters (A,B) within the same row indicate significant differences between drying methods for the same acid (*p* < 0.05), A. Oven-dried pectin. B. Freeze-dried pectin, according to Tukey’s multiple range test. Data are expressed as mean ± SD.

**Table 3:** Molecular weight (MW_p_) quantification of lemon by-product pectin following acid-assisted extraction using citric acid, sulphuric acid (H_2_SO_4_), and hydrochloric acid (HCl).

Acid Conventional Extraction	Pectin Drying Method	Molecular Weight (MW_p_)	
		First Peak (kDa)	Second Peak (kDa)
Citric Acid	Oven-dried	185.34 ± 1.40 ^aA^	3.82 ± 0.01 ^aA^
	Freeze-dried	198.00 ± 2.00 ^aA^	4.76 ± 0.13 ^aB^
HCl	Oven-dried	184.02 ± 2.32 ^aA^	
	Freeze-dried	235.47 ± 1.79 ^bB^	
H_2_SO_4_	Oven-dried	226.40 ± 2.86 ^bA^	3.30 ± 0.02 ^aA^
	Freeze-dried	233.80 ± 1.77 ^bA^	3.34 ± 0.08 ^bA^

MW_p_: Molecular weight at maximum peak. Different lowercase letters (a,b) within the same column indicate significant differences among acids for the same drying method (*p* < 0.05). Different uppercase letters (A,B) within the same row indicate significant differences between drying methods for the same acid (*p* < 0.05), A. Oven-dried pectin. B. Freeze-dried pectin, according to Tukey’s multiple range test. Data are expressed as mean ± SD.

**Table 4:** Degree of esterification (DE) calculated through titration and FTIR spectroscopy, and the degree of methylation (DM) of lemon by-product pectin.

Acid Conventional Extraction	Pectin Drying Method	Degree of Esterification (DE%)		Degree of Methylation (DM%) *	
		Titration	FTIR Spectroscopy	Titration	FTIR Spectroscopy
Citric Acid	Oven-dried	69.28 ± 1.43 ^aA^	76.33 ± 1.84 ^aA^	11.43 ± 0.23 ^aA^	12.44 ± 0.30 ^aA^
	Freeze-dried	77.04 ± 1.99 ^aB^	81.35 ± 4.76 ^aA^	12.60 ± 0.32 ^aB^	13.26 ± 0.78 ^aA^
HCl	Oven-dried	88.39 ± 1.01 ^bA^	76.18 ± 3.86 ^aA^	14.43 ± 0.16 ^bA^	12.42 ± 0.63 ^aA^
	Freeze-dried	85.65 ± 0.60 ^bB^	78.84 ± 1.24 ^aA^	14.01 ± 0.10 ^bB^	12.85 ± 0.20 ^aA^
H_2_SO_4_	Oven-dried	87.83 ± 0.66 ^bA^	75.50 ± 0.69 ^aA^	14.35 ± 0.11 ^bA^	12.31 ± 0.11 ^aA^
	Freeze-dried	81.00 ± 0.21 ^cB^	78.94 ± 0.07 ^aA^	13.22 ± 0.04 ^cB^	12.87 ± 0.01 ^aA^
Commercial Citrus Pectin (Sigma)	-	85.25 ± 1.94	73.49 ± 2.06	13.90 ± 0.32	11.98 ± 0.34

Different lowercase letters (a–c) within the same column indicate significant differences among acids for the same drying method (*p* < 0.05). Different uppercase letters (A,B) within the same row indicate significant differences between drying methods for the same acid (*p* < 0.05), A. Oven-dried pectin. B. Freeze-dried pectin, according to Tukey’s multiple range test. Data are expressed as mean ± SD. DM (%) * was estimated using the conversion proposed by Han et al. (2024) [30]. This calculation assumes a theoretical maximum methoxy content of 16.3%, corresponding to 100% esterification, and was adopted in this study for estimation purposes (Equation (3)).

**Table 5:** Thermal parameters of lemon by-products pectin samples as determined by differential scanning calorimetry (DSC).

Acid Conventional Extraction	Pectin Drying Method	Endothermic Peak		Exothermic Peak	
		Δ*H*_m_ (J/g)	T_m_ (°C)	Δ*H*_d_ (J/g)	T_d_ (°C)
Citric Acid	Oven-dried	193.6 ± 16.6 ^aA^	155.7 ± 1.6 ^aA^	114.0 ± 8.7 ^aA^	251.9 ± 6.4 ^aA^
	Freeze-dried	256.1 ± 13.6 ^aB^	145.9 ± 1.7 ^aA^	134.4 ± 3.0 ^aB^	258.1 ± 0.8 ^aB^
HCl	Oven-dried	207.4 ± 2.7 ^aA^	150.7 ± 12.4 ^aA^	135.2 ± 3.5 ^bA^	254.0 ± 3.3 ^abA^
	Freeze-dried	217.3 ± 13.4 ^bA^	147.3 ± 9.6 ^aA^	159.1 ± 11.3 ^bB^	250.8 ± 1.6 ^bA^
H_2_SO_4_	Oven-dried	254.1 ± 13.7 ^bA^	145.9 ± 4.5 ^aA^	134.8 ± 8.0 ^bA^	258.1 ± 1.7 ^bA^
	Freeze-dried	228.0 ± 6.3 ^bB^	152.6 ± 11.7 ^aA^	155.5 ± 4.0 ^bB^	251.6 ± 0.6 ^bB^
Commercial Citrus Pectin (Sigma)	-	239.1 ± 5.5	178.5 ± 8.7	190.7 ± 10.9	251.5 ± 0.3

Δ*H*_m_ (J/g), enthalpy of melting; T_m_ (°C), melting temperature; Δ*H*_d_ (J/g), enthalpy of degradation; T_d_ (°C), degradation temperature. Different lowercase letters (a,b) within the same column indicate significant differences among acids for the same drying method (*p* < 0.05). Different uppercase letters (A,B) within the same row indicate significant differences between drying methods for the same acid (*p* < 0.05), A. Oven-dried pectin. B. Freeze-dried pectin, according to Tukey’s multiple range test. Data are expressed as mean ± SD.

## Data Availability

The original contributions presented in this study are included in the article. Further inquiries can be directed to the corresponding author(s).

## References

[1] Freitas C.M.P., Coimbra J.S.R., Souza V.G.L., Sousa R.C.S. (2021). Structure and Applications of Pectin in Food, Biomedical, and Pharmaceutical Industry: A Review. Coatings.

[2] Wang X., Chen Q., Lü X. (2014). Pectin Extracted from Apple Pomace and Citrus Peel by Subcritical Water. Food Hydrocoll..

[3] Zannini D., Dal Poggetto G., Malinconico M., Santagata G., Immirzi B. (2021). Citrus Pomace Biomass as a Source of Pectin and Lignocellulose Fibers: From Waste to Upgraded Biocomposites for Mulching Applications. Polymers.

[4] Dixit S.S., Muruganandam L., Ganesh Moorthy I. (2025). Pectin from Fruit Peel: A Comprehensive Review on Various Extraction Approaches and Their Potential Applications in Pharmaceutical and Food Industries. Carbohydr. Polym. Technol. Appl..

[5] Sultana N. (2023). Biological Properties and Biomedical Applications of Pectin and Pectin-Based Composites: A Review. Molecules.

[6] Gawkowska D., Cybulska J., Zdunek A. (2018). Structure-Related Gelling of Pectins and Linking with Other Natural Compounds: A Review. Polymers.

[7] Assifaoui A., Hayrapetyan G., Gallery C., Agoda-Tandjawa G. (2024). Exploring Techno-Functional Properties, Synergies, and Challenges of Pectins: A Review. Carbohydr. Polym. Technol. Appl..

[8] Said N.S., Olawuyi I.F., Lee W.Y. (2023). Pectin Hydrogels: Gel-Forming Behaviors, Mechanisms, and Food Applications. Gels.

[9] Lara-Espinoza C., Carvajal-Millán E., Balandrán-Quintana R., López-Franco Y., Rascón-Chu A. (2018). Pectin and Pectin-Based Composite Materials: Beyond Food Texture. Molecules.

[10] Van Rooyen B., De Wit M., Van Niekerk J. (2024). Pectin and Alginate Functional Biopolymers: Factors Influencing Structural Composition, Functional Characteristics and Biofilm Development. Coatings.

[11] Abdalla G., Mussagy C.U., Sant’Ana Pegorin Brasil G., Scontri M., da Silva Sasaki J.C., Su Y., Bebber C., Rocha R.R., de Sousa Abreu A.P., Goncalves R.P. (2023). Eco-Sustainable Coatings Based on Chitosan, Pectin, and Lemon Essential Oil Nanoemulsion and Their Effect on Strawberry Preservation. Int. J. Biol. Macromol..

[12] Tumbarski Y., Petkova X., Todorova M., Ivanov I., Deseva I., Mihaylova D., Ibrahim S.A. (2020). Effects of Pectin-Based Edible Coatings Containing a Bacteriocin of Bacillus Methylotrophicus BM47 on the Quality and Storage Life of Fresh Blackberries. Ital. J. Food Sci..

[13] Valdés A., Burgos N., Jiménez A., Garrigós M.C. (2015). Natural Pectin Polysaccharides as Edible Coatings. Coatings.

[14] Maftoonazad N., Ramaswamy H.S. (2019). Application and Evaluation of a Pectin-Based Edible Coating Process for Quality Change Kinetics and Shelf-Life Extension of Lime Fruit (*Citrus aurantifolium*). Coatings.

[15] Rajapaksha D.C., Edirisinghe S.L., Nikapitiya C., Dananjaya S., Kwun H.-J., Kim C.-H., Oh C., Kang D.-H., De Zoysa M. (2020). Spirulina Maxima Derived Pectin Nanoparticles Enhance the Immunomodulation, Stress Tolerance, and Wound Healing in Zebrafish. Mar. Drugs.

[16] Eivazzadeh-Keihan R., Noruzi E.B., Aliabadi H.A.M., Sheikhaleslami S., Akbarzadeh A.R., Hashemi S.M., Gorab M.G., Maleki A., Cohan R.A., Mahdavi M. (2022). Recent Advances on Biomedical Applications of Pectin-Containing Biomaterials. Int. J. Biol. Macromol..

[17] Guo H., Du Y., Gao H., Liao Y., Liu H., Wu D., Gan R., Gao H. (2024). Isolation, Purification, Degradation of Citrus Pectin and Correlation between Molecular Weight and Their Biological Properties. LWT.

[18] Qi T., Ren J., Li X., An Q., Zhang N., Jia X., Pan S., Fan G., Zhang Z., Wu K. (2023). Structural Characteristics and Gel Properties of Pectin from Citrus Physiological Premature Fruit Drop. Carbohydr. Polym..

[19] Norulfairuz D., Zaidel A., Rashid J., Hazirah N. (2017). Extraction and Characterization of Pectin from Dragon Fruit (*Hylocereus polyrhizus*) Peels. Chem. Eng. Trans..

[20] Kamal M.M., Kumar J., Mamun M.A.H., Ahmed M.N.U., Shishir M.R.I., Mondal S.C. (2021). Extraction and Characterization of Pectin from Citrus Sinensis Peel. J. Biosyst. Eng..

[21] Akhter M.J., Sarkar S., Sharmin T., Mondal S.C. (2024). Extraction of Pectin from Powdered Citrus Peels Using Various Acids: An Analysis Contrasting Orange with Lime. Appl. Food Res..

[22] Code of Federal Regulations. https://www.ecfr.gov/current/title-21/chapter-I/subchapter-B/part-184.

[23] Dirpan A., Deliana Y., Ainani A.F., Irwan, Bahmid N.A. (2024). Exploring the Potential of Pectin as a Source of Biopolymers for Active and Intelligent Packaging: A Review. Polymers.

[24] Magalhães D., Rodrigues C.V., Botella-Martinez C., Muñoz-Tebar N., Angel Pérez-Álvarez J., Viuda-Martos M., Teixeira P., Pintado M. (2025). Lemon Dietary Fibre-Based Powder as a Promising Ingredient for the Food Industry: Enhancing Mortadella Nutritional Quality. Foods.

[25] Pasandide B., Khodaiyan F., Mousavi Z., Hosseini S.S. (2018). Pectin Extraction from Citron Peel: Optimization by Box–Behnken Response Surface Design. Food Sci. Biotechnol..

[26] Latimer G.W. (2012). Official Methods of Analysis of AOAC International.

[27] Garna H., Mabon N., Nott K., Wathelet B., Paquot M. (2006). Kinetic of the Hydrolysis of Pectin Galacturonic Acid Chains and Quantification by Ionic Chromatography. Food Chem..

[28] Campos D.A., Coscueta E.R., Vilas-Boas A.A., Silva S., Teixeira J.A., Pastrana L.M., Pintado M.M. (2020). Impact of Functional Flours from Pineapple By-Products on Human Intestinal Microbiota. J. Funct. Foods..

[29] Panwar D., Panesar P.S., Chopra H.K. (2023). Ultrasound-Assisted Extraction of Pectin from Citrus Limetta Peels: Optimization, Characterization, and Its Comparison with Commercial Pectin. Food Biosci..

[30] Han C., Zhao X., Yang L., Yao M., Zhang J., He Q., Liu J., Liu L. (2024). Extraction and Structural Analysis of Sweet Potato Pectin and Characterization of Its Gel. Polymers.

[31] Manrique G.D., Lajolo F.M. (2002). FT-IR Spectroscopy as a Tool for Measuring Degree of Methyl Esterification in Pectins Isolated from Ripening Papaya Fruit. Postharvest Biol. Technol..

[32] Dambuza A., Rungqu P., Oyedeji A.O., Miya G.M., Kuria S.K., Hosu S.Y., Oyedeji O.O. (2024). Extraction, Characterization, and Antioxidant Activity of Pectin from Lemon Peels. Molecules.

[33] Panwar D., Panesar P.S., Chopra H.K. (2024). Green Extraction of Pectin from Citrus Limetta Peels Using Organic Acid and Its Characterization. Biomass Convers. Biorefin..

[34] Rivas-Navia D., Jiménez-López N., Torrens E., Mbakidi J.-P., Bouquillon S., Bengoa C. (2026). Protein Extraction from Alphitobius Diaperinus Using Choline-Based Amino Acid Ionic Liquids. Food Bioproc. Technol..

[35] Ha S.M.L., de Bruijn W.J.C., Vreeke G.J.C., Bruins M.E., Nieuwland M., van der Goot A.J., Keppler J.K. (2025). Protein Extraction from Oat: Isoelectric Precipitation and Water-Based Extraction. Future Foods.

[36] Maskey B., Dhakal D., Pradhananga M., Shrestha N.K. (2018). Extraction and Process Optimization of Bael Fruit Pectin. Food Sci. Nutr..

[37] Rahman M.S., Khan S.S., Ahmed M.W., Jony M.E., Das P.C., Uddin M.B. (2023). Extraction of Pectin from Elephant Apple and Pomelo Fruit Peels: Valorization of Fruit Waste towards Circular Economy. Food Chem. Adv..

[38] Ubieko E.A., Onugwu A.L., Ogbonna J.D., Okoye E., Nwakile C.D., Attama A.A. (2023). Phytochemistry of the Extracted Pectin from Citrus Sinensis Fruit Peels. Univers. J. Pharm. Res..

[39] Dambuza A., Rungqu P., Oyedeji A.O., Miya G., Hosu S., Oriola A.O., Oyedeji O.O. (2025). Characterization and Antioxidant Activity of Pectin from Lemon Peels. Open Chem..

[40] El Fihry N., El Mabrouk K., Eeckhout M., Schols H.A., Filali-Zegzouti Y., Hajjaj H. (2022). Physicochemical and Functional Characterization of Pectin Extracted from Moroccan Citrus Peels. LWT.

[41] Li H., Li Z., Wang P., Liu Z., An L., Zhang X., Xie Z., Wang Y., Li X., Gao W. (2024). Evaluation of Citrus Pectin Extraction Methods: Synergistic Enhancement of Pectin’s Antioxidant Capacity and Gel Properties through Combined Use of Organic Acids, Ultrasonication, and Microwaves. Int. J. Biol. Macromol..

[42] Wani K.M., Uppaluri R.V.S. (2023). Characterization of Pectin Extracted from Pomelo Peel Using Pulsed Ultrasound Assisted Extraction and Acidic Hot Water Extraction Process. Appl. Food Res..

[43] Muñoz-Almagro N., Rico-Rodriguez F., Villamiel M., Montilla A. (2018). Pectin Characterisation Using Size Exclusion Chromatography: A Comparison of ELS and RI Detection. Food Chem..

[44] Liu N., Yang W., Li X., Zhao P., Liu Y., Guo L., Huang L., Gao W. (2022). Comparison of Characterization and Antioxidant Activity of Different Citrus Peel Pectins. Food Chem..

[45] Karim R., Nahar K., Zohora F.T., Islam M.M., Bhuiyan R.H., Jahan M.S., Shaikh M.A.A. (2022). Pectin from Lemon and Mango Peel: Extraction, Characterisation and Application in Biodegradable Film. Carbohydr. Polym. Technol. Appl..

[46] Monsoor M.A., Kalapathy U., Proctor A. (2001). Improved Method for Determination of Pectin Degree of Esterification by Diffuse Reflectance Fourier Transform Infrared Spectroscopy. J. Agric. Food Chem..

[47] Akachat B., Himed L., Salah M., D’Elia M., Zomorroda S., Ali R., Rastrelli L., Barkat M. (2025). Optimization of Pectin Extraction from Lemon Peels Using Hydrochloric Acid and Response Surface Methodology. Appl. Food Res..

[48] Zioga M., Tsouko E., Maina S., Koutinas A., Mandala I., Evageliou V. (2022). Physicochemical and Rheological Characteristics of Pectin Extracted from Renewable Orange Peel Employing Conventional and Green Technologies. Food Hydrocoll..

[49] Singhal S., Deka S.C., Koidis A., Hulle N.R.S. (2025). Standardization of Extraction of Pectin from Assam Lemon (*Citrus limon* Burm f.) Peels Using Novel Technologies and Quality Characterization. Biomass Convers. Biorefin..

[50] Yu Y., Lu P., Yang Y., Ji H., Zhou H., Chen S., Qiu Y., Chen H. (2024). Differences in Physicochemical Properties of Pectin Extracted from Pomelo Peel with Different Extraction Techniques. Sci. Rep..

[51] Ponmurugan K., Al-Dhabi N.A., Maran J.P., Karthikeyan K., Moothy I.G., Sivarajasekar N., Manoj J.J.B. (2017). Ultrasound Assisted Pectic Polysaccharide Extraction and Its Characterization from Waste Heads of Helianthus Annus. Carbohydr. Polym..

[52] Piacenza E., Presentato A., Alduina R., Scurria A., Pagliaro M., Albanese L., Meneguzzo F., Ciriminna R., Chillura Martino D.F. (2022). Cross-Linked Natural IntegroPectin Films from Citrus Biowaste with Intrinsic Antimicrobial Activity. Cellulose.

[53] Liang Y., Yang Y., Zheng L., Zheng X., Xiao D., Wang S., Ai B., Sheng Z. (2022). Extraction of Pectin from Passion Fruit Peel: Composition, Structural Characterization and Emulsion Stability. Foods.

[54] Xiao L., Ye F., Zhou Y., Zhao G. (2021). Utilization of Pomelo Peels to Manufacture Value-Added Products: A Review. Food Chem..

[55] Hua X., Wang K., Yang R., Kang J., Zhang J. (2015). Rheological Properties of Natural Low-Methoxyl Pectin Extracted from Sunflower Head. Food Hydrocoll..

[56] Jong S.H., Abdullah N., Muhammad N. (2023). Rheological Characterization of Low Methoxyl Pectin Extracted from Durian Rind. Carbohydr. Polym. Technol. Appl..

[57] Gamonpilas C., Krongsin J., Methacanon P., Goh S.M. (2015). Gelation of Pomelo (*Citrus maxima*) Pectin as Induced by Divalent Ions or Acidification. J. Food Eng..

